# The Distribution of Asterosaponins, Polyhydroxysteroids and Related Glycosides in Different Body Components of the Far Eastern Starfish *Lethasterias fusca*

**DOI:** 10.3390/md17090523

**Published:** 2019-09-06

**Authors:** Roman S. Popov, Natalia V. Ivanchina, Alla A. Kicha, Timofey V. Malyarenko, Boris B. Grebnev, Valentin A. Stonik, Pavel S. Dmitrenok

**Affiliations:** G.B. Elyakov Pacific Institute of Bioorganic Chemistry, Far Eastern Branch of Russian Academy of Sciences, 159 Prospect 100-letiya Vladivostoku, Vladivostok 690022, Russia (R.S.P.) (N.V.I.) (A.A.K.) (T.V.M.) (B.B.G.) (V.A.S.)

**Keywords:** starfish, *Lethasterias fusca*, asterosaponins, polyhydroxysteroids, glycosides, body components, distribution

## Abstract

Glycoconjugated and other polar steroids of starfish have unique chemical structures and show a broad spectrum of biological activities. However, their biological functions remain not well established. Possible biological roles of these metabolites might be indicated by the studies on their distribution in the organism–producer. In order to investigate the localization of polar steroids in body components of the Far Eastern starfish *Lethasterias fusca*, chemical constituents of body walls, gonads, stomach, pyloric caeca, and coelomic fluid were studied by nanoflow liquid chromatography/mass spectrometry with captive spray ionization (nLC/CSI–QTOF–MS). It has been shown that the levels of polar steroids in the studied body components are qualitatively and quantitatively different. Generally, the obtained data confirmed earlier made assumptions about the digestive function of polyhydroxysteroids and protective role of asterosaponins. The highest level of polar steroids was found in the stomach. Asterosaponins were found in all body components, the main portion of free polyhydroxysteroids and related glycosides were located in the pyloric caeca. In addition, a great inter-individual variability was found in the content of most polar steroids, which may be associated with the peculiarities in their individual physiologic status.

## 1. Introduction

Starfish are characterized by a high content of polar, frequently glycoconjugated steroids. Polar steroid compounds from starfish have put together an important class of biologically active marine metabolites, including steroids bearing four to nine hydroxyl groups, related polyhydroxysteroid mono-, bi- and triosides, and oligoglycosides known as asterosaponins [1,2,3,4,5,6]. Structural diversity of these metabolites is significant. Several hundred new individual natural compounds were discovered from different species of Asteroidea up to date.

Glycoconjugated polyhydroxysteroids usually have one or two monosaccharide units in steroid nucleus, side chain or both steroid nucleus and side chain. Aglycones of asterosaponins are proved to be Δ^9(11)^-3β,6α-dihydroxysteroids with a sulfate group at C-3, while their oligosaccharide chains, affixed to C-6 of aglycone, contain four to six sugar units with one or two branchings. The side chains of asterosaponin aglycones and their oligosaccharide fragments show significant structural diversity [1,2,3,4,5,6].

Polar steroids from starfish demonstrate cytotoxic, antibacterial, antiviral, antifungal, anticancer, anti-inflammatory, analgesic, and neuritogenic effects [1,2,3,4,5,6]. At the same time, the knowledge about their biological functions is limited. To understand biological roles of these substances in the organism–producer, accurate information of the polar steroid distribution is helpful because it is assumed that the biological roles of the steroid metabolites may be related to organ and tissue distribution. In addition, the data concerning the localization of these metabolites in animals might be useful in the targeted isolation of new natural compounds.

Previously, the studies on the asterosaponin distribution were based on the determination of sugar composition and the hemolytic activities of the corresponding extracts due to high hemolytic properties of asterosaponins, while polyhydroxysteroids and related glycosides show only weak or moderate hemolytic activity. Asterosaponins were found in both inner organs and body walls of starfish *Asterias amurensis*, *Asterias rubens*, and *Marhtasterias glacialis* [7,8,9]. Analysis of the asterosaponin fractions isolated from aboral and oral body walls, stomach, gonads, and pyloric caeca of the starfish *Leptasterias polaris* by liquid chromatography demonstrated the presence of characteristic mixtures of asterosaponins in the different body parts [10]. Recently, a combination of matrix-assisted laser desorption/ionization mass spectrometry (MALDI–MS), MALDI–MS imaging and liquid chromatography coupled to mass spectrometry (LC/MS) was applied to study the diversity, body distribution and localization of asterosaponins in *A. rubens* [11,12]. Asterosaponins from aboral and oral body walls, pyloric caeca, gonads, and stomach from four animals were extracted and analyzed separately, allowing comparisons to be made between body parts and between individuals [11]. Asterosaponins were found in all body parts of all individual starfish, but each organ was characterized by a distinctive set of asterosaponins and their concentrations varied significantly among organs as well as among individuals. Pyloric caeca were shown to be organs presenting the lowest asterosaponin concentrations. Although extracts of body walls and gonads showed high asterosaponin concentrations, significant differences between individuals were noted. MALDI–MS imaging was used to clarify inter- and intra-organ spatial distributions of asterosaponins that had been early found in *A. rubens* extracts [12]. This approach, performed at different spatial resolutions, revealed the complicated character of distributions of asterosaponins and showed that some asterosaponins are located not only inside the body walls of the starfish but also within the outer mucus layer, where they probably protect the animals against predators. Thus, the body distribution of asterosaponins indicates that the participation in the defense of starfish against predatory fish can be the main biological function of these glycosides. In addition, it is suggested that asterosaponins could be involved in reproduction processes and interspecific chemical signaling [1,6].

The distribution of free sterols, polyhydroxysteroids and steroid glycosides in aboral and oral body walls, gonads, stomach, and pyloric caeca of the starfish *Patiria* (=*Asterina*) *pectinifera* has been studied [13]. It was shown that all these body components contained asterosaponins. However, polyhydroxysteroids and glycosides of polyhydroxysteroids were located mainly in the stomach and pyloric caeca [13]. It was suggested that these metabolites are involved in the digestion of food based on the presence of these compounds in digestive organs of the starfish and their structural resemblance to certain bile alcohols. It has also been established that the concentration of polyhydroxylated steroids and polyhydroxysteroid glycosides in stomach and pyloric caeca of *P. pectinifera* depends on the season and is related to the periods of active feeding [14,15].

The Far Eastern starfish *Lethasterias fusca* has been recently studied by our group and 14 polar steroid compounds, including three polyhydroxysteroids, six glycosides of polyhydroxysteroids, and five asterosaponins, were isolated [16,17]. The cancer-preventive activity of the asterosaponins was investigated. It has been shown that lethasterioside A demonstrates a considerable inhibition of the human breast T-47D (97%), melanoma RPMI-7951 (90%), and colorectal carcinoma HCT-116 (90%) cell colony formations in a soft agar clonogenic assay [16]. Recently, we have applied a nanoflow liquid chromatography/tandem mass spectrometry with captive spray ionization (nLC/CSI–QTOF–MS/MS) for the profiling and characterization of the polar steroid metabolites of *L. fusca*. In total, 207 steroid compounds, including 106 asterosaponins, six native aglycones of asterosaponins, 81 polyhydroxysteroid glycosides, and 14 non-glycoconjugated polyhydroxysteroids, were found. According to the obtained data, exact structures of twenty steroids were determined and tentative structures for previously undescribed steroid compounds were proposed [18].

Herein, we describe the application of nLC/CSI–QTOF–MS for the profiling of purified fractions of polar steroids from body walls, coelomic fluid, gonads, stomach, and pyloric caeca to establish the content of these metabolites in different body components of the starfish *L. fusca*.

## 2. Results and Discussion

Previously, we have studied polar steroid metabolites of the starfish *L. fusca* by nanoflow liquid chromatography coupled with a captive spray ionization time-of-flight tandem mass spectrometer (nLC/CSI–QTOF–MS/MS). As a result, 207 polar steroids, including 81 glycosides of polyhydroxysteroids, 14 polyhydroxylated steroids, 106 asterosaponins, and six native aglycones of asterosaponins were detected. Although exact structures cannot be deduced from mass spectrometry data only, a detailed fragmentation analysis with accurate mass measurement allowed for characterizing and to proposing tentative structures for the detected compounds. Among the identified asterosaponins, new compounds with unusual monosaccharide units and new aglycone types were found. Polyhydroxysteroids and related glycosides were found in both sulfated and non-sulfated forms and demonstrated great structural diversity [18].

### 2.1. Distribution of Polar Steroids in Body Components of the Starfish L. fusca

The present study was undertaken to establish the distribution of previously detected polar steroid compounds in different body components of the starfish *L. fusca*. For this purpose, we separately extracted polar steroids from body walls (BW), gonads (G), stomach (S), and pyloric caeca (PC) from five animals. Starfish coelomic fluids (CF), which are in contact with all internal organs, were also collected and analyzed. To obtain a purified fraction of polar steroid compounds from complicated ethanolic extracts, the two-stage liquid–liquid extraction followed by desalting by solid-phase extraction was used. The quality of extraction of polar steroid metabolites was controlled by ESI MS and LC/ESI MS at every stage (data not shown). Finally, polar steroid metabolites from different body components of the starfish *L. fusca* were analyzed qualitatively and semi-quantitatively by nLC/CSI–QTOF–MS. Based on the previous results, the mass spectra were recorded in negative ion mode. Some typically obtained base-peak chromatograms are shown in Figure S1. Amounts of every compound were semi-quantified using lethasterioside A as a reference standard for asterosaponins, fuscaside A as a reference standard for sulfated polyhydroxysteroid glycosides and sulfated polyhydroxysteroids and 5α-cholestan-3β,4β,6α,7α,8,15β,16β,26-octaol as a reference standard for non-sulfated polyhydroxysteroid compounds and non-sulfated polyhydroxysteroid glycosides and are shown as ng/g of the wet weight of the organs (Table S4).

**Figure 1:**
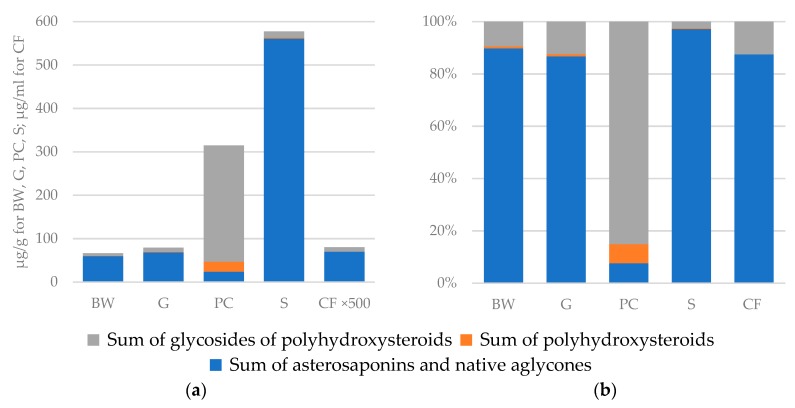
(**a**) concentrations of asterosaponins, polyhydroxysteroids, and related glycosides in different organs and coelomic fluid of the starfish *L. fusca* (μg/g wet weight of the organs for body walls (BW), gonads (G), pyloric caeca (PC) and stomach (S) and μg/mL for coelomic fluid (CF); CF values multiplied 500-fold); (**b**) ratios of concentrations of asterosaponins, polyhydroxysteroids, and related glycosides in different organs and coelomic fluid of the starfish *L. fusca*.

The profiling of polar steroids from various body components showed that the distribution of these compounds is qualitatively and quantitatively different (Table 1, Table S4, Figure 1). The maximal content of the sum of all polar steroids was observed in the stomach when compared with other body components of *L. fusca* (577.5 μg/g of wet weight, Table 1). The main part of polar steroids in the stomach was proved to be asterosaponins and native aglycones of asterosaponins (97% of all polar steroids content in the stomach). It can be assumed that asterosaponins in the stomach have a protective function against predators, the same as in body walls. It is known that starfish have the so-called “external” nutrition, they evert the stomach from the organism’s body and engulf the food. Probably, at such moments, the protection of this organ is especially important. At the same time, it is known that asterosaponins exhibit antimicrobial properties [1,2,3,4,5,6] and can protect the organism of a starfish from pathogenic microorganisms coming from food. Toxic properties of asterosaponins also can help starfish immobilize or kill living creatures in food.

Polar steroids content in the pyloric caeca was 314.4 μg/g. The main compounds in PC were glycosides of polyhydroxysteroids (85%); asterosaponins and polyhydroxysteroids presented a less significant part (both about 7%) (Table 1, Figure 1). These data are in good agreement with the previously hypothesized digestive role of polyhydroxysteroids and their related glycosides [13]. The body walls and gonads contained smaller concentrations of polar steroid compounds (66.5 and 78.9 μg/g, respectively). Major steroid constituents of both organs were identified as asterosaponins (90% for BW and 87% for G), a less substantial part was presented by polyhydroxysteroid glycosides (9% for BW and 12% for G) (Table 1, Figure 1).

Profiling of the coelomic fluid obtained from all five individuals showed that the content of polar steroids in all CF samples was minimal among analyzed samples. The average content of polar steroids in CF was 0.16 μg/mL of coelomic fluid, which is below 0.02% of the sum of all polar steroid content in the starfish (Table 1, Figure 1). Most of the compounds were either detected in trace amounts or not detected. Only 29 compounds showed a concentration higher than 1 ng/mL of coelomic fluid. Among them were 23 asterosaponins, four sulfated glycosides, and the maximum concentrations were shown by two native aglycones of asterosaponins (3-*O*-sulfothornasterol A (**205**) (20.9 ng/mL) and 3-*O*-sulfo-24,25-dihydromarthasterone (**207**) (10.9 ng/mL)) (Table S4). Due to the low content of the studied compounds, the CF samples were excluded for further analysis of the distribution of polar steroids.

### 2.2. Distribution of Asterosaponins

**Figure 2:**
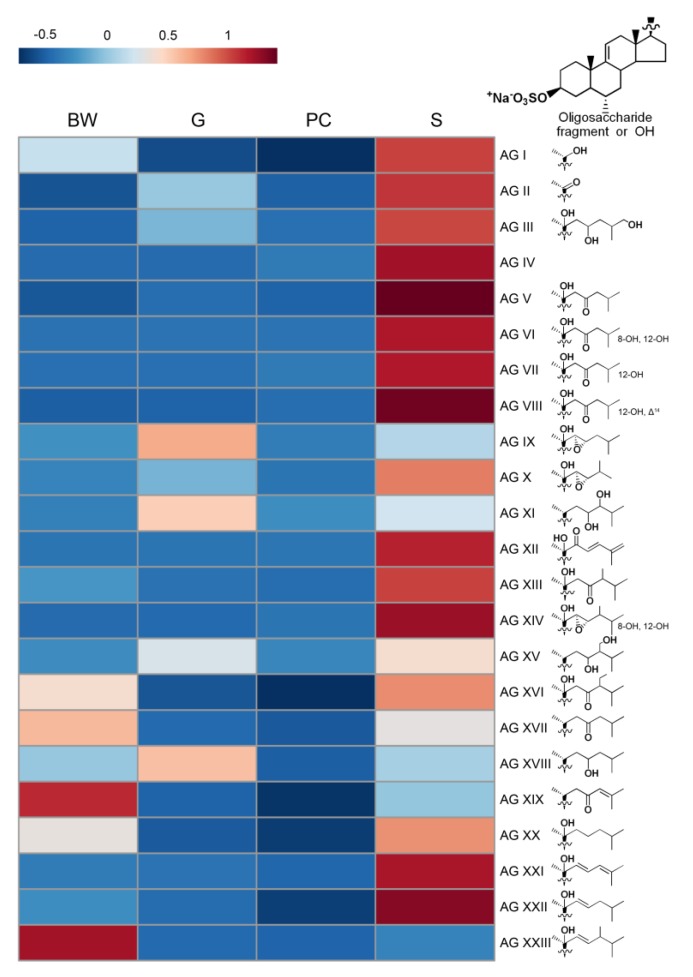
Heatmap demonstrating relative content of asterosaponins in different organs of the starfish *L. fusca*. Each row represents the sum content of asterosaponins with the same aglycone type (AG I–AG XXIII). Color bars represent auto-scaled relative abundances of asterosaponin groups, going from less (blue) to more (red).

Previously, 112 asterosaponins, including 66 pentaosides, 28 hexaosides, 7 triosides, five “shortened” asterosaponins with one-monosaccharide units at C-6 and six native aglycones of asterosaponins were found and structurally characterized in *L. fusca* [18]. According to the proposed structures of aglycones, all the detected asterosaponins of *L. fusca* were divided into twenty-three groups (I—XXIII) according to the types of aglycones (AG I—AG XXIII) (structure of aglycones given in Figure 2).

Asterosaponins were detected in all studied body parts. In the body walls, gonads, and stomach asterosaponins were the main class of detected polar steroids, but the stomach had a maximal concentration of these compounds (561.1, 68.4, and 59.7 μg/g in the stomach, gonads, and body walls, respectively). In addition, each organ was characterized by its specific mixture of asterosaponins. The majority of asterosaponins were present at a maximum concentration in the stomach; other asterosaponins were more characteristic of the body walls or gonads. The content of forty-two asterosaponins was found to be statistically different between the various organs by ANOVA and Tukey’s post hoc test (Table 2, Table S4); the content of other seventy asterosaponins showed a large inter-individual variability.

Often, the localization of asterosaponins was associated with the type of aglycone (Figure 2). At the same time, the structure of the oligosaccharide chains did not affect the distribution of asterosaponins. All asterosaponins with aglycones having additional hydroxy groups in steroid nucleus (AG VI, AG VII, AG VIII, and AG XIV), asterosaponins **16** (AG IV) and **65** (AG XII), most of the asterosaponins with aglycones AG V, AG XXI, and AG XXII as well as their native aglycones (**205** (3-*O*-sulfothornasterol A (AG V)), **174** (AG VIII), and **178** (AG VII) were found mainly in the stomach. On the contrary, it was established that concentrations of asterosaponins with aglycone AG XIX and asterosaponin **194** (AG XXIII) were significantly higher in the body walls. Asterosaponins with AG I were shown to be characteristic of body walls and stomach, and asterosaponins with AG II (3-*O*-sulfoasterone) were characteristic of gonads and stomach. Levels of other compounds, belonging to this class, did not show significant differences and had a large inter-individual variability. Asterosaponins with AG XIII, AG XVI, AG XVII, and AG XX were distributed between body walls and stomach, and asterosaponins with AG III, AG IX, AGX, AG XI, AG XV, and AG XVIII were distributed between body walls and gonads.

Previous investigations of the asterosaponins distribution have also shown that these compounds presented in all starfish organs and each organ had a characteristic mixture of asterosaponins. For example, in *P. pectinifera*, asterosaponins were found in maximal concentrations in the aboral and oral body walls and gonads [13]. In addition, in *A. rubens*, the highest concentrations of asterosaponins were measured in the body walls and gonads [11,12]. Otherwise, it was found that asterosaponin content in the stomach of *L. polaris* was several times greater than in the body walls and pyloric caeca [19]. Our results also indicate that the asterosaponins content prevails in the stomach rather than in the body walls or gonads. The fact that some asterosaponins are found only in one organ and not in others may indicate quite specific biological function of individual asterosaponins in starfish.

Thus, our data along with previously obtained results demonstrate the presence of asterosaponins in all starfish body components. The fact that toxic triterpene glycosides, which are considered to be part of chemical defense system in sea cucumbers, were also found in all body components of sea cucumbers [20,21,22] may support the notion of the protective function of asterosaponins.

### 2.3. Distribution of Polyhydroxysteroids and Glycosides of Polyhydroxysteroids

The highest concentrations of both non-sulfated and sulfated polyhydroxysteroid compounds were observed in extracts of the pyloric caeca (23.1 μg/g) (Table 1, Figure 1). The content of non-sulfated polyhydroxysteroids 5α-cholestane-3β,4β,6α,7α,8,15α,16β,26-octaol (**4**), 5α-cholestane-3β,6α,7α,8,15α,16β,26-heptaol (**11**), and 5α-cholestane-3β,4β,6α,7α,8,15β,16β,26-octaol (**15**) and sulfated polyhydroxysteroids 5α-cholestane-3β,4,6,7,8,15α,16β,26-octaol 6-*O*-sulfate (**63**), 5α-cholestane-3β,6,8,15α,16β,26-hexaol 6-*O*-sulfate (**117**), 5α-cholestane-3β,6,8,15β,16β,26-hexaol 6-*O*-sulfate (**133**), 5α-cholestane-3β,6,8,15,24-pentaol 24-*O*-sulfate (**136**), 5α-ergost-22-ene-3β,6,7,8,15α,16β,26-heptaol 6-*O*-sulfate (**146**), and 5α-ergost-22-ene-3β,6,8,15,16β,26-hexaol 6-*O*-sulfate (**153**) were found to be statistically different between the pyloric caeca and other samples by ANOVA with Tukey’s post hoc test (Table 3). Polyhydroxysteroids were about 10-fold less concentrated in the stomach (about 2 μg/g) than in the pyloric caeca. However, it should be noted that samples S#2, S#3, and S#4 contained large concentrations of sulfated polyhydroxysteroid **30** (the structure was not assigned), while all other polyhydroxysteroids showed maximum concentrations in the pyloric caeca samples. In addition,, stomach extracts showed a significant amount of 5α-cholestane-3β,6,8,15α,16β,26-hexaol 6-*O*-sulfate (**117**), 5α-cholestane-3β,6,8,15β,16β,26-hexaol 6-*O*-sulfate (**133**), and 5α-ergost-22-ene-3β,6,8,15,16β,26-hexaol 6-*O*-sulfate (**153**). The analysis of the body walls and gonads showed significantly lower concentrations of polyhydroxysteroid compounds (0.5 and 0.7 μg/g, respectively).

Analysis of the starfish extracts from different body components showed that the pyloric caeca are characterized by maximal level of non-sulfated and sulfated glycosides of polyhydroxysteroids among all samples. Concentrations of four non-sulfated and thirty-four sulfated glycosides were found to be significantly higher in the pyloric caeca than in other organs from the ANOVA analysis (Table 3). Concentrations of other glycosides of this structural group were also higher in the pyloric caeca samples; however, there was a large inter-individual variability. For example, the extract of the pyloric caeca of animal #2 (PC#2) was characterized by a high concentrations of non-sulfated 3-*O*-Xyl-24-*O*-Xyl-glycosylated 5α-cholest-pentaol derivatives—fuscaside B (**5**), distolasteroside D_1_ (**10**), and distolasteroside D_2_ (**14**)—and by low concentrations of 28-*O*-pentosyl-5α-ergostane-3β,6,8,15,16β,28-hexaol (7) and 24-*O*-hexosyl-5α-cholestane-3β,6,8,15β,24-pentaol (**12**). On the contrary, sample PC#5 had high concentrations of **7** and **12** and a low concentration of **5**, **10**, and **14**. Sample PC#4 had a maximal concentration of 24-*O*-pentosyl-5α-cholestane-3β,6,8,15α,16β,24-hexaol (**1**), 24-*O*-hexosyl-5α-cholestane-3β,6,7,8,15α,16β,24-heptaol (**2**), pycnopodioside A (**18**), and desulfated minutoside A (**21**); all of these compounds are 24-*O*-glycosylated polyhydroxysteroids.

For sulfated glycosides, a similar distribution was observed. While all the glycosides were more concentrated in the pyloric caeca samples, each animal was characterized by its own ratio of these compounds. The PC#2 is characterized by a high content of most of glycosides having pentose unit at C-3 and sulfated monosaccharide unit at the side chain: 3-*O*-pentosyl-24-*O*-sulfohexosyl-5α-cholestane-3β,6,8,15,24-pentaol (**46**), 3-*O*-pentosyl-28-*O*-sulfohexosyl-5α-ergostane-3β,6,8,15,28-pentaol (**57**), 3-*O*-pentosyl-26-*O*-sulfohexosyl-27-nor-5α-ergost-22-ene-3β,6,8,15,26-pentaol (**69**), 3-*O*-pentosyl-28-*O*-sulfohexosyl-5α-ergost-22-ene-3β,6,8,15,28-pentaol (**85**), 3-*O*-pentosyl-24-*O*-sulfopentosyl-5α-cholestane-3β,6,8,15,24-pentaol (**92**), 3-*O*-pentosyl-26-*O*-sulfohexosyl-5α-ergost-22-ene-3β,6,8,15,26-pentaol (**94**), 3-*O*-pentosyl-28-*O*-sulfopentosyl-5α-ergost-20(22)-ene-3β,6,8,15,28-pentaol (**114**), and 3-*O*-pentosyl-24-*O*-methylsulfopentosyl-5α-cholestane-3β,6,8,15,24-pentaol (**145**). PC#5 had a high concentration of certain pentaol derivatives with monosaccharide units at the side chains: 24-*O*-hexosyl-5α-cholestane-3β,6,8,15,24-pentaol 3-*O*-sulfate (**48**), 28-*O*-sulfohexosyl-5α-ergost-20(22)-ene-3β,6,8,15,28-pentaol (**103**), 28-*O*-[sulfohexosyl-hexosyl]-5α-ergost-22-ene-3β,6,8,15,28-pentaol (**119**), 28-*O*-sulfohexosyl-5α-ergostane-3β,6,8,15,28-pentaol (**126**), 24-*O*-sulfopentosyl-5α-cholestane-3β,6,8,15,24-pentaol (**155**), 29-*O*-sulfohexosyl-5α-stigmastane-3β,6,8,15,29-pentaol (**185**), and 28-*O*-sulfopentosyl-5α-ergost-20(22)-ene-3β,6,8,15,28-pentaol (**188**). PC#5 also had a minimal concentration of glycosylated hexaols among all pyloric caeca samples while samples PC#1 and PC#2 had a higher content of glycosylated hexaols. In addition, sample PC#5 contained minimal concentrations of glycosides of polyhydroxysteroids with stigmastane side chains (except glycoside **185**).

In the stomach, body walls and gonads, non-sulfated glycosides presented in trace amounts. Sulfated glycosides were found in the other organs but only in small quantities. Localization of polyhydroxysteroids and glycosides of polyhydroxysteroids in the *L. fusca* pyloric caeca confirmed data previously obtained by Kicha et al. on the distribution of polar steroids of *P. pectinifera*, demonstrated that polyhydroxysteroids and polyhydroxysteroid glycosides were localized mainly in the stomach and pyloric caeca [13,14,15]. It was suggested that the location of these compounds in starfish digestive organs along with their definite structural resemblance to bile alcohols of hagfishes and amphibians and their ability to solubilize a lipid suspension connected with roles of these compounds in digestion processes. The observed high inter-individual variability may be associated with the biogenesis of these compounds. It is known that only a part of cholestane steroids is synthesized de novo in the starfish [23,24]. Other cholestane steroids, as well as all steroids with stigmastane and ergostane side chains, are biosynthesized from dietary phytosterols and dietary cholesterol [25]. Therefore, different ratios of polyhydroxysteroids and related glycosides in individual animals may be related to their diet.

## 3. Materials and Methods

### 3.1. Chemicals

Water (LC/MS grade) and acetonitrile (UHPLC grade) were purchased from Panreac (Barcelona, Spain), methanol (HPLC grade) was purchased from J.T. Baker (Deventer, the Netherlands). All other chemicals were of analytical grade or equivalent. Lethasterioside A, fuscaside A, and 5α-cholestan-3β,4β,6α,7α,8,15β,16β,26-octaol isolated early from the starfish *L. fusca* were used as standards of polar steroid glycosides. Structures of these compounds were established using different methods including high-resolution NMR [16,17].

### 3.2. Animal Material

Individuals of *L. fusca* (order Forcipulatida, family Asteriidae) were collected at the coastal area of the Posyet Gulf, the Sea of Japan, in August 2017, from a depth of 1–3 m. Identification of the species was carried out by B.B. Grebnev (G.B. Elyakov Pacific Institute of Bioorganic Chemistry of the Far Eastern Branch of Russian Academy of Sciences (PIBOC FEB RAS), Vladivostok, Russia). All animals were sexually mature and ranged in diameter from 10 to 18 cm; the identification of sex of the animals was not performed. The voucher specimen No. PIBOC-2017-08-LF is preserved in the collection of PIBOC FEB RAS.

### 3.3. Sample Preparation and Solid-Phase Extraction (SPE)

Five freshly caught animals were rapidly dissected and separated into body walls (BW), gonads (G), stomach (S) and pyloric caeca (PC). The wet weights of the starfish, as well as weights of collected body parts, are listed in Table S1. In addition, the coelomic fluid (CF) of the individuals was obtained by puncturing at the arm tip and collected by gravity into separate cold tubes. BW, G, S, and PC were undergoing the triple extraction with ten folds volumes of ethanol during 10 h. The extracts from different body components as well as CF were filtered and evaporated in vacuo. For removing lipid contaminations, dried samples were dissolved with a solvent combination of chloroform: methanol: water (CHCl_3_/MeOH/H_2_O 8:4:3, *v*/*v*/*v*) to a final dilution 30-fold in relation to the weight of the dried sample. After centrifugation (2000 *g* for 10 min) 100 μL of the upper water-methanolic layer were transferred to another vial and subjected to the second liquid–liquid extraction with a solvent combination of CHCl_3_/MeOH/H_2_O (1000:450:250 μL). The 400 μL of the upper layer were transferred to another vial, evaporated in vacuo, dissolved in 50% methanol in water (*v*/*v*, 150 μL) and were subjected to the solid-phase extraction for desalting. Sorbent of SPE cartridges (Bond Elut C18 Cartridges, 100 mg/1 mL, Agilent Technologies, Santa Clara, CA, USA) was moistened by 3 mL of acetonitrile (ACN) and equilibrated with 3 mL of 0.1% formic acid (FA) in water. The 100 μL sample was loaded into the SPE cartridge by drops. After washing the cartridge with 0.5 mL of 0.1% FA, polar steroid metabolites were eluted with 1.5 mL of 100% ACN, evaporated and dissolved in 200 μL 50% ACN. Samples were centrifuged (15,000 *g* for 10 min) and supernatant was placed in 200 μL glass micro insert (Agilent Technologies, Santa Clara, CA, USA) in an autosampler vial (2 mL) (Agilent Technologies, Santa Clara, CA, USA) for LC/MS.

### 3.4. LC/MS Analysis

All samples were subjected to nLC/CSI–QTOF–MS analysis using an UltiMate 3000 RSLCnano System (Dionex, Sunnyvale, CA, USA) connected to a Bruker Impact II Q-TOF mass spectrometer (Bruker Daltonics, Bremen, Germany) equipped with a CaptiveSpray ionization source (Bruker Daltonics, Bremen, Germany). Analysis conditions were similar to what was described previously [18]. Acclaim PepMap RSLC column (75 μm × 150 mm, nanoViper, C18, 2 μm, 100 A; Thermo Scientific, City, US State abbrev. if applicable, Country) with a cartridge-based trap column µ-Precolumn (300 μm × 5 mm, C18, 5 μm, 100 A; Thermo Scientific) was applied for separation of polar steroids at 40 °C, and the injection volume was 0.2 μL. Chromatographic separation was done with water containing 0.1% FA as solvent A and ACN containing 0.1% FA as solvent B using the following gradient profile: 0–5 min 34% B; 5–20 min ramping to 58% B; 20–70 min ramping to 80% B; 70–71 min ramping to 99% B; 71–80 min hold at 99% B; 80–81 min return to 34% B; and equilibration at 34% B during 15 min; flow rate of 400 nl/min. The mass spectrometer was operated in a negative ion mode using the following parameters: mass range 100–2000 *m*/*z*; capillary voltage, 1300 V; the drying gas temperature, 150 °C; the drying gas flow rate, 3 l/min. ESI-L Low Concentration Tuning Mix (Agilent Technologies, Santa Clara, CA, USA) and hexakis(1H,1H,3H-tetrafluoropropoxy)phosphazine (966.0007 *m*/*z* in negative mode; Agilent Technologies, Santa Clara, CA, USA) were used for calibration and lock-mass calibration, respectively. The otofControl (ver. 4.0, Bruker Daltonics, Bremen, Germany) and DataAnalysis Software (ver. 4.3, Bruker Daltonics, Bremen, Germany) were using for obtaining and analyzing of MS data.

A series of four consecutive injections of a pooled sample of *L. fusca* were run prior to the sample analysis to conditions of the chromatographic column. The analysis of replicate injections showed little retention time shift following this conditioning procedure. Samples were analyzed in random order. Quality control samples (pooled samples of starfish) and blank samples (50% ACN) were analyzed after every three samples throughout the batch analysis in order to check the performance of data acquisition and to ensure reproducibility. Method blanks demonstrated no carry-over between sample runs.

The raw spectra were converted to mzML files by the open-source msConvert tool of the ProteoWizard library [26]. Data preprocessing was performed using MZmine 2.40 [27]. Applied parameters are given as supplementary material (Table S2). The peak detection batch step was carried out via a targeted peak detection module using the peak list file containing retention times and *m*/*z* values of previously detected steroid metabolites of *L. fusca* [18] (Table S3). Sulfated steroid metabolites were detected as [M − Na]^−^ ions, non-sulfated polyhydroxysteroids and related glycosides were detected as [M − H]^−^ and [M + FA]^−^ ions. Resulted data (*m*/*z* values, retention times and peak area) were exported into a csv file. Compound identification was performed by comparing their retention time, elemental composition and MS/MS spectrum with those data obtained previously [18]. A mass accuracy tolerance between the measured mass and the theoretical mass calculated from the molecular formula did not exceed 3 ppm. Numbers of detected compounds were assigned according to compound numbers from our previous work (Table S3).

### 3.5. Semi-Quantitative Analysis of Detected Polar Steroid Compounds

For semi-quantitative analysis, we used lethasterioside A as a reference standard for asterosaponins (*R*^2^ = 0.9922), fuscaside A as a reference standard for sulfated polyhydroxysteroid glycosides and sulfated polyhydroxysteroids (*R*^2^ = 0.9939) and 5α-cholestan-3β,4β,6α,7α,8,15β,16β,26-octaol as a reference standard for non-sulfated polyhydroxysteroid compounds and non-sulfated polyhydroxysteroid glycosides (*R*^2^ = 0.9888). Standard solutions at concentrations of 0.1, 1.0, 2.5, and 5.0 μg/mL were used for building calibration curves (Figures S2–S4). All experiments were carried out at least three times, LC/MS conditions are identical to those described above. As a result, the amounts of detected compounds were calculated through calibration curves. Results are shown in Table S4 as concentration in ng/g wet weight of animal organs.

Statistical analysis (ANOVA followed by Tukey’s HSD (honestly significant difference) test of multiple comparisons (α = 0.05)) was performed using Metaboanalyst 4.0 (www.metaboanalyst.ca, free software updated and maintained by Xia Lab at McGill University [28]). Data were auto-scaled prior to ANOVA tests. To avoid false-positive results, multiple comparisons were compensated for using false discovery rate (FDR) calculations [29], and FDRs were estimated using the *q*-value method [30]. A *q*-value < 0.05 was considered statistically significant.

## 4. Conclusions

The distribution of polar steroid compounds in different body components of the Far Eastern starfish *L. fusca* was investigated using a modern nLC/CSI–QTOF–MS technique. Comparison of the sum of polar steroid content and individual asterosaponins, polyhydroxysteroids and related glycosides from the body walls, coelomic fluid, gonads, stomach, and pyloric caeca was performed. It was shown that the distribution of individual asterosaponins, polyhydroxysteroids, and polyhydroxysteroid glycosides is qualitatively and quantitatively different. The toxic asterosaponins were found in all organs of the starfish. The maximal concentration of the sum of all polar steroids was observed in the stomach and most of them were asterosaponins and native aglycones of asterosaponins. The comparison of the content of individual asterosaponins in different organs of the starfish probably suggests different biological roles of these metabolites in the starfishes. This may be due to the toxic, protective or antimicrobial properties of these compounds. The main part of glycosides of polyhydroxysteroids was located in the pyloric caeca and this confirmed the digestive function of these steroids in starfishes. At the same time, the levels of these steroids can vary greatly depending on the individual. This observed high inter-individual variability may be associated with different physiological statuses of the animals and partly with the biogenesis of some these compounds from dietary steroids.

## Figures and Tables

**Table 1:** Concentrations of asterosaponins, polyhydroxysteroids, and related glycosides in different organs and coelomic fluid of the starfish *L. fusca* (μg/g wet weight of the organs for body walls, gonads, pyloric caeca and stomach and μg/mL for coelomic fluid).

	Asterosaponins and Native Aglycones	Polyhydroxysteroids	Glycosides of Polyhydroxysteroids	Total
Body walls (μg/g)	59.7	0.5	6.3	66.5
Gonads (μg/g)	68.4	0.7	9.8	78.9
Pyloric caeca (μg/g)	23.8	23.1	267.5	314.4
Stomach (μg/g)	561.1	1.9	14.5	577.5
Coelomic fluid (μg/mL)	0.14	-	0.02	0.16

**Table 2:** Asterosaponins that display significant differences (*q*-value < 0.05) between contents in the various body parts of the starfish *L. fusca* (content is given as a mean ± SD ng/g wet weight of the organs).

N	Content in Different Organs (Mean ± SD, ng/g)				Proposed Structures
	BW	G	PC	S	
**3**	27.5 ± 23.5	0.7 ± 0.9	0.8 ± 1	6.2 ± 1.5	Hex–dHex–Hex–Xyl(-Qui)–Qui–AG I
**16**	0.1 ± 0.2	0 ± 0	3.8 ± 3.5	87.7 ± 74.5	dHex–dHex–Qui(-Qui)–Hex–AG IV
**17**	50.1 ± 53.2	39.7 ± 24	79.3 ± 61.6	658.1 ± 232.1	Hex–dHex–Hex–Xyl(-Qui)–Qui–AG II
**32**	0.8 ± 0.8	3.5 ± 2.5	7.4 ± 3.4	818 ± 552.4	dHex–dHex–Qui(-Qui)–DXU–AG VI
**33**	45.4 ± 65.8	295.6 ± 526.5	20.3 ± 17.6	2749.1 ± 1759	dHex–dHex–Qui(-Qui)–DXU–AG II
**34**	93.2 ± 58.6	10.4 ± 6.5	13.8 ± 6.7	48.5 ± 32.5	AG I
**40**	23.9 ± 25.3	12.9 ± 18.7	18.3 ± 16.1	615.5 ± 394.5	Hex–dHex–Hex–Xyl(-Qui)–Qui–AG VII
**41**	0.9 ± 0.6	5.7 ± 9.6	5.8 ± 6.1	325.3 ± 204.6	Hex–dHex–Hex–Xyl(-Qui)–Qui–AG VIII
**44**	64.4 ± 70.9	8.9 ± 8.4	5.6 ± 4	350.2 ± 240.5	Hex–dHex–Hex–Xyl(-Qui)–Hex–AG V
**51**	86.1 ± 168.1	26.4 ± 22.3	45.4 ± 15.4	1059.8 ± 733.7	Qui–Xyl–Qui–AG I
**60**	80.7 ± 102.3	52 ± 39.6	67.3 ± 59.3	1232 ± 639.6	dHex–Hex–Xyl(-Qui)–Hex–AG V
**65**	0.5 ± 0.3	1.2 ± 2.1	3.9 ± 2.8	375.3 ± 370	dHex–dHex–Qui(-Qui)–Hex–AG XII
**70**	34.8 ± 15.4	39.4 ± 18.5	24 ± 11.3	2805.1 ± 929.5	dHex–dHex–Glc(-Qui)–Hex–AG V
**74**	4.8 ± 3.3	12 ± 6.4	11.7 ± 5.2	1468.7 ± 949.4	dHex–dHex–Qui(-Qui)–DXU–AG VIII
**77**	13 ± 4.9	30.6 ± 25.6	23.3 ± 8.2	2888.6 ± 1744.3	dHex–dHex–Qui(-Qui)–DXU–AG VII
**78**	2468.3 ± 2319.4	1881.1 ± 1119	953.2 ± 857.2	23,431.5 ± 7708.7	Hex–dHex–Hex–Xyl(-Qui)–Qui–AG V
**88**	77.5 ± 66.8	110.5 ± 52.2	33.4 ± 25.3	4674.9 ± 1961	Hex–dHex–dHex–Glc(-Qui)–Qui–AG V
**90**	0.2 ± 0.2	0 ± 0.1	2.6 ± 2.5	83.8 ± 67.6	dHex–dHex–Glc(-Qui)–Qui–AG XIV
**93**	104 ± 125.9	121.9 ± 83.6	71.4 ± 61	4978 ± 4835.3	dHex–dHex–Qui(-Qui)–Hex–AG V
**105**	8.8 ± 10.7	11.6 ± 12	10.1 ± 5.5	789.8 ± 563.5	dHex–dHex–Glc(-Qui)–Hex–AG XIII
**106**	29.7 ± 24.6	21.4 ± 12.8	39.8 ± 31.9	566 ± 187.1	dHex–dHex–Hex–Xyl(-Qui)–Qui–AG V
**111**	304.4 ± 169.6	153.5 ± 38	111.4 ± 65.9	44,57.6 ± 3242.3	dHex–Hex–Glc(-Qui)–Qui–AG V
**112**	53.5 ± 51.3	16.2 ± 15.1	7.5 ± 5.7	655.2 ± 516.7	Hex–dHex–Hex–Xyl(-Qui)–Hex–AG XVII
**118**	831.6 ± 848.4	2816.5 ± 1624.1	2521.9 ± 2733.1	10,972.3 ± 4393.7	Fuc–Gal–Xyl(-Qui)–Qui–AG V (thornasteroside A)
**122**	2553.5 ± 1926.5	6991.5 ± 3200.9	3934.5 ± 2011.3	173,398.3 ± 148,397.4	Fuc–Qui–Glc(-Qui)–Qui–AG V (luidiaquinoside)
**123**	2846.4 ± 2424.5	162.9 ± 111.1	42.8 ± 46.9	333.7 ± 264.9	Hex–dHex–Hex–Xyl(-Qui)–Qui–AG XX
**129**	37.4 ± 27.4	68.1 ± 40.1	25.7 ± 12.6	2677.6 ± 1160.6	Hex–dHex–dHex–Qui(-Qui)–Qui–AG V
**135**	437.3 ± 207.6	2343.4 ± 2062	273.3 ± 196.3	64,570.3 ± 23,737.3	dHex–dHex–Qui(-Qui)–DXU–AG V
**150**	1249.7 ± 1349	1098.9 ± 860.2	224 ± 215.9	28,498.5 ± 24,555.7	dHex–dHex–Qui(-Qui)–Hex–AG XXI
**152**	399.9 ± 462.7	931 ± 1346	114 ± 78.6	6581 ± 1828.8	dHex–dHex–Glc(-Qui)–Hex–AG XXII
**159**	1114.2 ± 860	305.4 ± 187.7	66.7 ± 57.1	364.7 ± 155.4	Fuc–Gal–Xyl(-Qui)–Qui–AG XVIII (lethasterioside B)
**174**	1.7 ± 2	13.6 ± 10.1	91.8 ± 61.2	818.9 ± 580.7	AG VIII
**175**	141.9 ± 90.5	355 ± 174.4	225.1 ± 120.2	7623.4 ± 3936.2	dHex–dHex–Qui(-Qui)–Qui–AG V
**178**	4.7 ± 5.2	25.3 ± 21.7	219.6 ± 166.5	1023.6 ± 609.4	AG VII
**180**	51 ± 40.9	725.5 ± 1061.2	95 ± 52	4869.7 ± 3668.5	dHex–dHex–Qui(-Qui)–(C_6_H_6_O_3_)–AG V
**182**	3440.1 ± 3370.9	1962.9 ± 1574	614.6 ± 567.6	36,581.4 ± 30,632.4	dHex–dHex–Qui(-Qui)–Hex–AG XXII
**186**	7.8 ± 5.5	633.3 ± 1139.3	50 ± 35.4	3777.4 ± 3427.8	dHex–dHex–Qui(-Qui)–(C_7_H_9_NO_4_)–AG V
**194**	295 ± 239.4	9.3 ± 5.8	4.7 ± 4.2	30.3 ± 38.5	Hex–dHex–Hex–Xyl(-Qui)–Qui–AG XXIII
**195**	1935.9 ± 1321.3	228.1 ± 163.7	14.6 ± 5.5	746.4 ± 802.9	dHex–dHex–Qui(-Qui)–DXU–AG XIX
**199**	4.8 ± 6.4	61.6 ± 76	68.6 ± 88.9	7221.9 ± 7112.6	dHex–dHex–Qui(-Qui)–(C_7_H_9_NO_4_)–AG V
**205**	1096.5 ± 566.1	10,113.8 ± 13,871.4	7737.1 ± 4085.7	4,5330.5 ± 21,263.7	3-*O*-sulfothornasterol A (AG V)
**207**	477.9 ± 255.2	154.4 ± 155.8	320.3 ± 99	664.4 ± 284.8	3-*O*-sulfo-24,25-dihydromarthasterone (AG XVII)

**Table 3:** Polyhydroxysteroids and related glycosides that display significant differences (q-value < 0.05) between contents in the various body parts of the starfish *L. fusca* (content is given as mean ± SD ng/g wet weight of the organs for BW, G, PC, and S).

	Content in Different Organs (Mean ± SD, ng/g)				Proposed Structures
	BW	G	PC	S	
**1**	1.1 ± 0.7	4.3 ± 7.3	369.8 ± 244.8	9.4 ± 7.6	24-*O*-pentosyl-5α-cholestane-3β,6,8,15α,16β,24-hexaol
**2**	1.5 ± 1.5	3.5 ± 5.2	421.5 ± 320.8	11.2 ± 7.2	24-*O*-hexosyl-5α-cholestane-3β,6,7,8,15α,16β,24-heptaol
**4**	0.1 ± 0.1	0.2 ± 0.5	123.8 ± 53.7	1.1 ± 0.4	5α-cholestane-3β,4β,6α,7α,8,15α,16β,26-octaol
**11**	0.4 ± 0.2	1 ± 1.7	325.9 ± 257.2	8.2 ± 6.4	5α-cholestane-3β,6α,7α,8,15α,16β,26-heptaol
**15**	2.2 ± 1.4	7.8 ± 11.3	1016.8 ± 582	15.3 ± 9.8	5α-cholestane-3β,4β,6α,7α,8,15β,16β,26-octaol
**18**	22.9 ± 18	70.6 ± 108.7	3276.3 ± 2782.6	167.5 ± 142.1	pycnopodioside A
**21**	17.8 ± 8.5	41.1 ± 51.9	2153.6 ± 1149.2	80.3 ± 53.5	desulfated minutoside A
**23**	26.3 ± 26.9	82.4 ± 148	1579.6 ± 1499.7	60.4 ± 54.8	3-*O*-pentosyl-5α-cholestane-3β,6,7,8,15,16β,26-heptaol 26-*O*-sulfate
**53**	10.6 ± 19.3	8.3 ± 8.3	164.8 ± 109	27.4 ± 42.9	24-*O*-sulfohexosyl-5α-cholestane-3β,6,8,15,16β,24-hexaol
**58**	64.8 ± 36.7	53.9 ± 62.7	2845.4 ± 1755.4	125.4 ± 81.2	-
**59**	49.8 ± 52.5	38.8 ± 40	2129.6 ± 1784.9	101.8 ± 62.5	3-*O*-pentosyl-5α-cholestane-3β,6,8,15,26-pentaol 26-*O*-sulfate
**63**	12.2 ± 12.5	16.9 ± 28.8	455.5 ± 388.1	22.8 ± 14	5α-cholestane-3β,4,6,7,8,15α,16β,26-octaol 6-*O*-sulfate
**71**	10.7 ± 6.9	4.5 ± 1.7	509.3 ± 373	19.8 ± 15.5	26-*O*-sulfohexosyl-27-nor-5α-ergost-22-ene-3β,6,8,15,16β,26-hexaol
**81**	177.8 ± 126.7	337.9 ± 578.6	7196.8 ± 3778.9	309.9 ± 243.4	24-*O*-pentosyl-5α-cholestane-3β,6,8,15,24-pentaol 3-*O*-sulfate
**83**	18.2 ± 18.4	8.9 ± 2.9	837.4 ± 721	35.5 ± 28	28-*O*-sulfohexosyl-5α-ergostane-3β,6,8,15,16β,28-hexaol
**91**	11.1 ± 10	5.4 ± 2.8	654.6 ± 581	20.4 ± 11.8	26-*O*-sulfohexosyl-27-nor-5α-ergost-22-ene-3β,6,8,15,16β,26-hexaol
**92**	35.2 ± 23.6	24.6 ± 32.1	924.1 ± 715.2	26.3 ± 9.1	3-*O*-pentosyl-24-*O*-sulfopentosyl-5α-cholestane-3β,6,8,15,24-pentaol
**94**	16.3 ± 14.1	19.1 ± 22.9	551.8 ± 333.7	23.8 ± 12.6	3-*O*-pentosyl-26-*O*-sulfohexosyl-5α-ergost-22-ene-3β,6,8,15,26-pentaol
**99**	10.4 ± 6.9	16.7 ± 21.8	582.9 ± 330	26.8 ± 14.5	28-*O*-sulfohexosyl-5α-ergost-20(22)-ene-3β,6,8,15,16β,28-hexaol
**108**	31.1 ± 18.9	53.7 ± 81.4	1191.2 ± 499.9	58.5 ± 26.7	3-*O*-pentosyl-24-*O*-methylsulfopentosyl-5α-cholestane-3β,6,8,15,24-pentaol
**109**	255.3 ± 194.8	213.7 ± 276.4	9046.4 ± 7355.2	476.4 ± 512.3	24-*O*-pentosyl-5α-cholest-22-ene-3β,6,8,15,24-pentaol 3-*O*-sulfate
**113**	68.3 ± 82.6	26.2 ± 17.1	2005.6 ± 1910.1	173.9 ± 243	26-*O*-sulfohexosyl-5α-ergost-22-ene-3β,6,8,15,16β,26-hexaol
**115**	33.9 ± 14.4	48.1 ± 68.6	1687.4 ± 1086.5	94.9 ± 64.4	24-*O*-sulfohexosyl-5α-cholest-20(22)-ene-3β,6,8,15,24-pentaol
**117**	21 ± 12.8	12.8 ± 7.8	994.9 ± 636	109 ± 89.2	5α-cholestane-3β,6,8,15α,16β,26-hexaol 6-*O*-sulfate
**120**	66.3 ± 74.4	24.5 ± 6.8	2870.3 ± 2423.8	122.3 ± 97.6	28-*O*-sulfohexosyl-5α-ergostane-3β,6,8,15,16β,28-hexaol
**125**	29.5 ± 13	30.4 ± 45.5	1280.1 ± 177.9	269.9 ± 315.4	-
**126**	63.6 ± 61.2	142 ± 260.4	2519.8 ± 1747.5	105.1 ± 107.1	28-*O*-sulfohexosyl-5α-ergostane-3β,6,8,15,28-pentaol
**131**	108.1 ± 73.9	124.3 ± 152.8	4377.2 ± 4373.3	194.2 ± 39.6	26-*O*-sulfohexosyl-27-nor-5α-ergost-20(22)-ene-3β,6,8,15,26-pentaol
**133**	21.1 ± 18.2	4.2 ± 1.4	620.1 ± 555.7	62 ± 59.2	5α-cholestane-3β,6,8,15β,16β,26-hexaol 6-*O*-sulfate
**136**	274.8 ± 165.5	549.3 ± 866.2	12,619.1 ± 6096.6	785.4 ± 489.5	5α-cholestane-3β,6,8,15,24-pentaol 24-*O*-sulfate
**138**	82 ± 41.9	185.6 ± 252.1	3811 ± 1281.6	122.3 ± 84.6	26-*O*-sulfohexosyl-27-nor-5α-ergost-20(22)-ene-3β,6,8,15,26-pentaol
**146**	10.8 ± 10.2	5.6 ± 4.1	584.6 ± 501.6	26.7 ± 25.3	5α-ergost-22-ene-3β,6,7,8,15α,16β,26-heptaol 6-*O*-sulfate
**147**	25.1 ± 22.7	14.4 ± 8.8	1053.4 ± 813.6	60 ± 48.5	29-*O*-sulfohexosyl-5α-stigmast-20(22)-ene-3β,6,8,15,16β,29-hexaol
**148**	14.9 ± 8.3	9.1 ± 6.5	777.4 ± 500.7	27.2 ± 19.5	28-*O*-sulfohexosyl-5α-ergost-22-ene-3β,6,8,15,16β,28-hexaol
**151**	76.2 ± 95.2	41.2 ± 14	3204.2 ± 2986.5	206.6 ± 204.7	28-*O*-sulfopentosyl-5α-ergost-20(22)-ene-3β,6,8,15,16β,28-hexaol
**153**	82.6 ± 58.9	40.5 ± 18.6	4829.9 ± 3939.8	342.7 ± 214.2	5α-ergost-22-ene-3β,6,8,15,16β,26-hexaol 6-*O*-sulfate
**154**	248.2 ± 182.6	488.2 ± 798.4	9881.2 ± 6654.5	629.1 ± 380.1	28-*O*-sulfohexosyl-5α-ergost-22-ene-3β,6,8,15,28-pentaol
**163**	70.5 ± 33.4	105.7 ± 164.8	3006.8 ± 503.1	172.5 ± 49.5	29-*O*-sulfohexosyl-5α-stigmastane-3β,6,8,15,16β,29-hexaol
**164**	210.1 ± 127.7	256.7 ± 404.1	6606.4 ± 2715.5	565.2 ± 205	26-*O*-sulfohexosyl-5α-ergost-22-ene-3β,6,8,15,26-pentaol
**165**	130.1 ± 93.8	86.5 ± 38.4	5780.8 ± 4339.3	315.8 ± 242.6	28-*O*-sulfohexosyl-5α-ergost-22-ene-3β,6,8,15,16β,28-hexaol
**171**	216.3 ± 130.9	419.9 ± 643.2	7547.9 ± 3816.2	478.3 ± 231.9	28-*O*-sulfohexosyl-5α-ergost-22-ene-3β,6,8,15,28-pentaol
**172**	18.1 ± 10	38.3 ± 54.7	1052 ± 570.4	123.6 ± 116.4	24-*O*-methylsulfopentosyl-5α-cholestane-3β,6,8,15,24-pentaol
**179**	276.4 ± 232.1	536.3 ± 873.4	10,891.4 ± 6548.5	681.7 ± 327.8	26-*O*-sulfohexosyl-5α-ergost-22-ene-3β,6,8,15,26-pentaol
**181**	53.5 ± 47.7	21.9 ± 4.9	2603.9 ± 2287.1	130.8 ± 110	29-*O*-sulfohexosyl-5α-stigmastane-3β,6,8,15,16β,29-hexaol
**183**	25 ± 27.3	10.4 ± 4.5	1081.5 ± 1033.7	67.5 ± 59.6	29-*O*-sulfohexosyl-5α-stigmast-22-ene-3β,6,8,15,16β,29-hexaol
**189**	24.3 ± 20.3	17.7 ± 11.1	1287.3 ± 919.2	65.8 ± 38.2	29-*O*-sulfohexosyl-5α-stigmast-22-ene-3β,6,8,15,16β,29-hexaol
**196**	335.5 ± 360.2	827.8 ± 1416.6	14,640.2 ± 14,245.6	1044.1 ± 925.3	24-*O*-sulfopentosyl-5α-cholest-22-ene-3β,6,8,15,24-pentaol

## Supplementary Materials

The following are available online at https://www.mdpi.com/1660-3397/17/9/523/s1, Table S1: Description of studied individuals of *L. fusca*, Figure S1: Typical nLC/CSI–QTOF–MS base-peak chromatograms of purified fractions of polar steroid compounds of different body components of the starfish *L. fusca* in negative ion mode: (a) body walls, sample BW#5; (b) coelomic fluid, sample CF#5; (c) gonads, sample G#5; (d) stomach, sample S#1; (e) pyloric caeca, sample PC#1, Table S2: Batch steps and parameters used for data preprocessing in MZmine, Table S3: Polar steroids of the starfish *L. fusca* detected by nLC/CSI–QTOF–MS, Figure S2. Calibration curve for fuscaside A (ion [M − Na]^−^ at *m*/*z* 795.38), Figure S3: Calibration curve for lethasterioside A (ion [M − Na]^−^ at *m*/*z* 1227.54), Figure S4: Calibration curve for 5α-cholestan-3β,4β,6α,7α,8,15β,16β,26-octaol (for the sum of ion intensities [M − H]^−^ at *m*/*z* 499.33 and [M + FA]^−^ at *m*/*z* 545.3), Table S4: Content of detected compounds in different organs and coelomic fluid of the starfish *L. fusca* (ng/g wet weight of the organs for BW, G, PC, and S and ng/mL for CF) and the result of statistical analysis (ANOVA followed by Tukey HSD test of multiple comparisons was performed for BW, G, PC, and S groups; *q*-value < 0.05 was considered statistically significant).
