# Supplementary material for: The Distribution of Asterosaponins, Polyhydroxysteroids and Related Glycosides in Different Body Components of the Far Eastern Starfish Lethasterias fusca
Source: Mar Drugs. 2019 Sep 6;17(9):523. doi: 10.3390/md17090523 (PMC6780307; doi:10.3390/md17090523)
Supplement: Supplementary file 1 [file marinedrugs-17-00523-s001.pdf]

Supplementary Materials: **The distribution of asterosaponins, polyhydroxysteroids and related glycosides in different body components of the Far Eastern starfish *Lethasterias fusca***

Roman S. Popov, Natalia V. Ivanchina, Alla A. Kicha, Timofey V. Malyarenko, Boris B. Grebnev, Valentin A. Stonik and Pavel S. Dmitrenok

Contents

Table S1. Description of studied individuals of *L. fusca*.

Figure S1. Typical nLC/CSI–QTOF–MS base-peak chromatograms of purified fractions of polar steroid compounds of different body components of the starfish *L. fusca* in negative ion mode: (a) body walls, sample BW#5; (b) coelomic fluid, sample CF#5; (c) gonads, sample G#5; (d) stomach, sample S#1; (e) pyloric caeca, sample PC#1.

Table S2. Batch steps and parameters used for data preprocessing in MZmine.

Table S3. Polar steroids of the starfish *L. fusca* detected by nLC/CSI–QTOF–MS.

Figure S2. Calibration curve for fuscaside A (ion $[M–Na]^-$ at *m/z* 795.38).

Figure S3. Calibration curve for lethasterioside A (ion $[M–Na]^-$ at *m/z* 1227.54).

Figure S4. Calibration curve for 5α-cholestan-3β,4β,6α,7α,8,15β,16β,26-octaol (for the sum of ion intensities $[M–H]^-$ at *m/z* 499.33 and $[M+FA]^-$ at *m/z* 545.3).

Table S4. Content of detected compounds in different organs and coelomic fluid of the starfish *L. fusca* (ng/g wet weight of the organs for BW, G, PC, and S and ng/ml for CF) and result of statistical analysis (ANOVA followed by Tukey HSD test of multiple comparisons was performed for BW, G, PC, and S groups; *q*-value < 0.05 was considered statistically significant).

Table S1. Description of studied individuals of *L. fusca*.

Individual	Animal size, mm	Animal weight, g	Wet weight of the organs, g				Volume of coelomic fluid, ml
			BW	G	PC	S	
1	178	286.2	163.76	3.04	67.80	3.69	15.0
2	153	220.3	85.39	0.70	32.37	3.26	24.0
3	128	103.6	53.63	-*	19.33	1.21	7.5
4	101	57.3	27.51	0.25	6.11	0.50	3.0
5	109	87.8	45.66	0.19	10.51	0.80	7.0

* Starfish #3 has undeveloped gonads. BW: body wall, G: gonads, PC: pyloric caeca, S: stomach

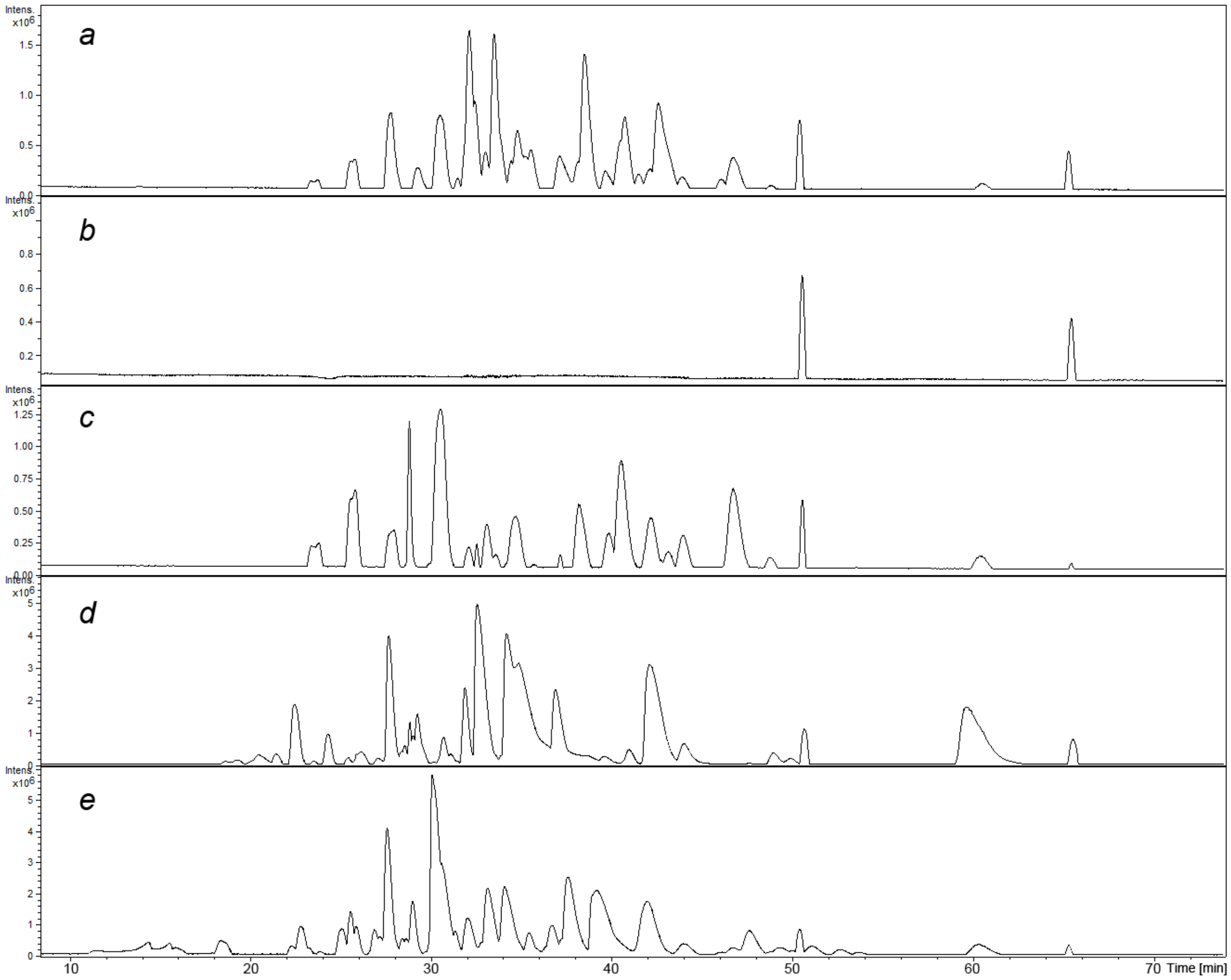

Figure S1. Typical nLC/CSI–QTOF–MS base-peak chromatograms of purified fractions of polar steroid compounds of different body components of the starfish *L. fusca* in negative ion mode: (*a*) body walls, sample BW#5; (b) coelomic fluid, sample CF#5; (c) gonads, sample G#5; (d) stomach, sample S#1; (e) pyloric caeca, sample PC#1.

Table S2 Batch steps and parameters used for data preprocessing in MZmine

Batch step	Module	Parameters	
Raw data import	-		
Raw data filtering	Crop filter	Retention time:	5 – 80 min
Chromatogram detection	Targeted peak detection	Noise level:	300
		m/z tolerance:	5 ppm
		Retention time tolerance:	3 %
Chromatogram deconvolution	Local minimum search	Chromatographic threshold:	85 %
		Search minimum in Rt range:	0.15
		Minimum absolute height:	1000
		Minimum ratio of peak top/edge:	2
		Peak duration range:	0.1 – 2.0 min
Normalization of retention time	Retention time normalizer	*m/z* tolerance:	3 ppm
		Retention time tolerance:	0.5 min
		Minimum standard intensity:	50000
Alignment	Join aligner	*m/z* tolerance:	5 ppm
		Retention time tolerance:	0.5 min
Gap filling	Same RT and m/z range gap filler	*m/z* tolerance:	5 ppm
Export to CSV			

Table S3. Polar steroids of the starfish *L. fusca* detected by nLC/CSI–QTOF–MS

No[a]	Rt (min)	Elemental composition	Calculated *m/z*	Measured m/z	Δ (ppm)	Ion type	Proposed structures
**1**	8.96	$C_{32}H_{56}O_{10}$	599.3801	599.3805	-0.7	$[M-H]^-$	24-*O*-pentosyl-5α-cholestane-3β,6,8,15α,16β,24-hexaol
			645.3856	645.3862	-0.9	$[M+FA]^-$	
**2**	9.15	$C_{33}H_{58}O_{12}$	645.3856	645.3857	-0.2	$[M-H]^-$	24-*O*-hexosyl-5α-cholestane-3β,6,7,8,15α,16β,24-heptaol
			691.391	691.3913	-0.4	$[M+FA]^-$	
**3**	12.69	$C_{56}H_{91}O_{32}SNa$	1307.522	1307.5223	-0.2	$[M-Na]^-$	Hex–dHex–Hex–Xyl(-Qui)–Qui–AG I
**4**	13.3	$C_{27}H_{48}O_{8}$	499.3276	499.3279	-0.6	$[M-H]^-$	5α-cholestane-3β,4β,6α,7α,8,15α,16β,26-octaol
			545.3331	545.3330	0.2	$[M+FA]^-$	
**5**	13.77	$C_{37}H_{64}O_{13}$	715.4274	715.4280	-0.8	$[M-H]^-$	fuscaside B
			761.4329	761.4333	-0.5	$[M+FA]^-$	
**6**	14.06	$C_{27}H_{43}O_{11}SNa$	575.2532	575.2535	-0.5	$[M-Na]^-$	Hex–AG I
**7**	14.2	$C_{33}H_{58}O_{10}$	613.3957	613.3962	-0.8	$[M-H]^-$	28-*O*-pentosyl-5α-ergostane-3β,6,8,15,16β,28-hexaol
			659.4012	659.4015	-0.5	$[M+FA]^-$	
**8**	14.39	$C_{57}H_{94}NO_{27}SNa$	1256.5739	1256.5743	-0.3	$[M-Na]^-$	dHex–dHex–Qui(-Qui)–($C_6H_9NO_3$)–AG III
**9**	14.67	$C_{27}H_{43}O_{11}SNa$	575.2532	575.2534	-0.3	$[M-Na]^-$	Hex–AG I
**10**	14.7	$C_{37}H_{64}O_{13}$	715.4274	715.4271	0.4	$[M-H]^-$	distolasteroside D_1
			761.4329	761.4331	-0.3	$[M+FA]^-$	
**11**	14.8	$C_{27}H_{48}O_{7}$	483.3327	483.3337	-2.1	$[M-H]^-$	5α-cholestane-3β,6α,7α,8,15α,16β,26-heptaol
			529.3382	529.3387	-0.9	$[M+FA]^-$	
**12**	14.93	$C_{33}H_{58}O_{10}$	613.3957	613.3963	-1.0	$[M-H]^-$	24-*O*-hexosyl-5α-cholestane-3β,6,8,15β,24-pentaol
			659.4012	659.4019	-1.1	$[M+FA]^-$	
**13**	15.66	$C_{49}H_{79}O_{26}SNa$	1115.4586	1115.4599	-1.2	$[M-Na]^-$	dHex–Pent–Xyl(-Qui)–Qui–AG I
**14**	15.85	$C_{37}H_{62}O_{13}$	713.4118	713.4124	-0.8	$[M-H]^-$	distolasteroside D_2
			759.2172	759.2172	0.0	$[M+FA]^-$	
**15**	16.02	$C_{27}H_{48}O_{8}$	499.3276	499.3284	-1.6	$[M-H]^-$	5α-cholestane-3β,4β,6α,7α,8,15β,16β,26-octaol
			545.3331	545.3337	-1.1	$[M+FA]^-$	
**16**	16.14	$C_{53}H_{85}O_{28}SNa$	1201.4964	1201.4966	-0.2	$[M-Na]^-$	dHex–dHex–Qui(-Qui)–Hex–AG IV
**17**	16.58	$C_{56}H_{89}O_{32}SNa$	1305.5063	1305.5068	-0.4	$[M-Na]^-$	Hex–dHex–Hex–Xyl(-Qui)–Qui–AG II
**18**	17.15	$C_{32}H_{56}O_{9}$	583.3852	583.3856	-0.7	$[M-H]^-$	pycnopodioside A
			629.3906	629.3918	-1.9	$[M+FA]^-$	
**19**	17.7	$C_{27}H_{48}O_{7}$	483.3327	483.3332	-1.0	$[M-H]^-$	5α-cholestane-3β,6α,7α,8,15β,16β,26-heptaol
			529.3382	529.3383	-0.2	$[M+FA]^-$	
**20**	17.72	$C_{50}H_{81}O_{26}SNa$	1129.4742	1129.4741	0.1	$[M-Na]^-$	dHex–Pent–Qui(-Qui)–Qui–AG I
**21**	18.17	$C_{32}H_{54}O_{9}$	581.3695	581.3704	-1.5	$[M-H]^-$	desulfated minutoside A
			627.375	627.3757	-1.1	$[M+FA]^-$	
**22**	18.85	$C_{50}H_{79}O_{27}SNa$	1143.4535	1143.4539	-0.3	$[M-Na]^-$	dHex–Hex–Xyl(-Qui)–Qui–AG II
**23**	18.86	$C_{32}H_{55}O_{14}SNa$	695.3318	695.3315	0.4	$[M-Na]^-$	3-*O*-pentosyl-5α-cholestane-3β,6,7,8,15,16β,26-heptaol 26-*O*-sulfate
**24**	19.04	$C_{27}H_{48}O_{6}$	467.3378	467.3375	0.6	$[M-H]^-$	5α-cholestane-3β,6α,8,15β,16β,26-hexaol
			513.3433	513.3428	1.0	$[M+FA]^-$	
**25**	19.07	$C_{57}H_{93}O_{30}SNa$	1289.5478	1289.5483	-0.4	$[M-Na]^-$	dHex–dHex–Glc(-Qui)–Qui–AG VI
**26**	19.16	$C_{27}H_{43}O_{10}SNa$	559.2582	559.2582	0.0	$[M-Na]^-$	dHex–AG I
**27**	19.35	$C_{51}H_{81}O_{27}SNa$	1157.4691	1157.4692	-0.1	$[M-Na]^-$	dHex–dHex–Glc(-Qui)–Qui–AG II
**28**	19.47	$C_{38}H_{61}O_{18}SNa$	837.3584	837.3582	0.2	$[M-Na]^-$	Qui–Xyl–Qui–AG I
**29**	20.01	$C_{49}H_{77}O_{26}SNa$	1113.4429	1113.4422	0.6	$[M-Na]^-$	dHex–Pent–Xyl(-Qui)–Qui–AG II
**30**	20.08	$C_{27}H_{45}O_{8}SNa$	529.2841	529.2832	1.7	$[M-Na]^-$	-
**31**	20.11	$C_{39}H_{63}O_{19}SNa$	867.369	867.3688	0.2	$[M-Na]^-$	Qui–Glc–Qui–AG I
**32**	20.2	$C_{57}H_{93}O_{30}SNa$	1289.5478	1289.5472	0.5	$[M-Na]^-$	dHex–dHex–Qui(-Qui)–DXU–AG VI
**33**	20.25	$C_{51}H_{81}O_{27}SNa$	1157.4691	1157.4690	0.1	$[M-Na]^-$	dHex–dHex–Qui(-Qui)–DXU–AG II
**34**	20.3	$C_{21}H_{33}O_{6}SNa$	413.2003	413.2003	0.0	$[M-Na]^-$	AG I
**35**	20.68	$C_{51}H_{81}O_{27}SNa$	1157.4691	1157.4694	-0.3	$[M-Na]^-$	dHex–dHex–Qui(-Qui)–DXU–AG II
**36**	21.17	$C_{51}H_{81}O_{27}SNa$	1157.4691	1157.4681	0.9	$[M-Na]^-$	dHex–Hex–Qui(-Qui)–Qui–AG II
**37**	21.55	$C_{50}H_{81}O_{26}SNa$	1129.4742	1129.4743	-0.1	$[M-Na]^-$	dHex–Pent–Qui(-Qui)–Qui–AG I
**38**	21.72	$C_{39}H_{61}O_{19}SNa$	865.3533	865.3536	-0.3	$[M-Na]^-$	Qui–Glc–Qui–AG II
**39**	22.12	$C_{39}H_{63}O_{18}SNa$	851.3741	851.3747	-0.7	$[M-Na]^-$	Qui–Qui–Qui–AG I
**40**	22.33	$C_{62}H_{101}O_{34}SNa$	1421.59	1421.5907	-0.5	$[M-Na]^-$	Hex–dHex–Hex–Xyl(-Qui)–Qui–AG VII
**41**	22.55	$C_{62}H_{99}O_{34}SNa$	1419.5744	1419.5748	-0.3	$[M-Na]^-$	Hex–dHex–Hex–Xyl(-Qui)–Qui–AG VIII
**42**	22.57	$C_{27}H_{43}O_{10}SNa$	559.2582	559.2581	0.2	$[M-Na]^-$	dHex–AG I
**43**	22.59	$C_{50}H_{79}O_{26}SNa$	1127.4586	1127.4595	-0.8	$[M-Na]^-$	dHex–Pent–Qui(-Qui)–Qui–AG II

No[a]	Rt (min)	Elemental composition	Calculated *m/z*	Measured m/z	Δ (ppm)	Ion type	Proposed structures
**44**	22.94	$C_{62}H_{101}O_{34}SNa$	1421.59	1421.5902	-0.1	[M–Na]⁻	Hex–dHex–Hex–Xyl(-Qui)–Hex–AG V
**45**	23.13	$C_{33}H_{57}O_{14}SNa$	709.3475	709.3480	-0.7	[M–Na]⁻	24-*O*-hexosyl-5α-cholestane-3β,6,8,15,16β,24-hexaol 3-*O*-sulfate
**46**	23.51	$C_{38}H_{65}O_{17}SNa$	825.3948	825.3951	-0.4	[M–Na]⁻	3-*O*-pentosyl-24-*O*-sulfohexosyl-5α-cholestane-3β,6,8,15,24-pentaol
**47**	23.56	$C_{51}H_{83}O_{26}SNa$	1143.4899	1143.4906	-0.6	[M–Na]⁻	dHex–dHex–Qui(-Qui)–Qui–AG I
**48**	23.8	$C_{33}H_{57}O_{13}SNa$	693.3525	693.3532	-1.0	[M–Na]⁻	24-*O*-hexosyl-5α-cholestane-3β,6,8,15,24-pentaol 3-*O*-sulfate
**49**	23.92	$C_{61}H_{99}O_{33}SNa$	1391.5795	1391.5805	-0.7	[M–Na]⁻	Hex–dHex–Hex–Xyl(-Qui)–Qui–AG X
**50**	24.01	$C_{51}H_{81}O_{26}SNa$	1141.4742	1141.4742	0.0	[M–Na]⁻	dHex–dHex–Qui(-Qui)–Qui–AG II
**51**	24.36	$C_{38}H_{61}O_{18}SNa$	837.3584	837.3588	-0.5	[M–Na]⁻	Qui–Xyl–Qui–AG I
**52**	24.38	$C_{51}H_{81}O_{26}SNa$	1141.4742	1141.4745	-0.3	[M–Na]⁻	dHex–dHex–Qui(-Qui)–Qui–AG II
**53**	24.5	$C_{33}H_{57}O_{14}SNa$	709.3475	709.3487	-1.7	[M–Na]⁻	24-*O*-sulfohexosyl-5α-cholestane-3β,6,8,15,16β,24-hexaol
**54**	24.64	$C_{27}H_{41}O_{10}SNa$	557.2426	557.2422	0.7	[M–Na]⁻	dHex–AG II
**55**	24.65	$C_{32}H_{55}O_{12}SNa$	663.342	663.3420	0.0	[M–Na]⁻	3-*O*-pentosyl-5α-cholestane-3β,6,8,15,26-pentaol 26-*O*-sulfate
**56**	25	$C_{33}H_{55}O_{14}SNa$	707.3318	707.3313	0.7	[M–Na]⁻	26-*O*-sulfohexosyl-5α-cholest-22-ene-3β,6,8,15,16β,26-hexaol
**57**	25.2	$C_{39}H_{67}O_{17}SNa$	839.4104	839.4101	0.4	[M–Na]⁻	3-*O*-pentosyl-28-*O*-sulfohexosyl-5α-ergostane-3β,6,8,15,28-pentaol
**58**	25.29	$C_{32}H_{53}O_{13}SNa$	677.3212	677.3209	0.4	[M–Na]⁻	-
**59**	25.35	$C_{32}H_{55}O_{12}SNa$	663.342	663.3415	0.8	[M–Na]⁻	3-*O*-pentosyl-5α-cholestane-3β,6,8,15,26-pentaol 26-*O*-sulfate
**60**	25.43	$C_{56}H_{91}O_{29}SNa$	1259.5372	1259.5361	0.9	[M–Na]⁻	dHex–Hex–Xyl(-Qui)–Hex–AG V
**61**	25.5	$C_{52}H_{80}NO_{26}SNa$	1166.4695	1166.4698	-0.3	[M–Na]⁻	dHex–dHex–Qui(-Qui)–($C_7H_9NO_4$)–AG II
**62**	25.52	$C_{55}H_{91}O_{27}SNa$	1215.5474	1215.5478	-0.3	[M–Na]⁻	dHex–Pent–Xyl(-Qui)–Qui–AG XI
**63**	25.68	$C_{27}H_{47}O_{11}SNa$	579.2845	579.2857	-2.1	[M–Na]⁻	5α-cholestane-3β,4,6,7,8,15α,16β,26-octaol 6-*O*-sulfate
**64**	25.74	$C_{37}H_{63}O_{16}SNa$	795.3842	795.3841	0.1	[M–Na]⁻	3-*O*-sulfopentosyl-24-*O*-pentosyl-5α-cholestane-3β,6,8,15,24-pentaol
**65**	25.97	$C_{57}H_{89}O_{28}SNa$	1253.5267	1253.5273	-0.5	[M–Na]⁻	dHex–dHex–Qui(-Qui)–Hex–AG XII
**66**	25.99	$C_{57}H_{93}O_{29}SNa$	1273.5529	1273.5537	-0.6	[M–Na]⁻	dHex–dHex–Glc(-Qui)–Qui–AG VII
**67**	26.11	$C_{63}H_{103}O_{34}SNa$	1435.6057	1435.6061	-0.3	[M–Na]⁻	Hex–dHex–Hex–Xyl(-Qui)–Hex–AG XIII
**68**	26.11	$C_{32}H_{55}O_{13}SNa$	679.3369	679.3379	-1.5	[M–Na]⁻	24-*O*-pentosyl-5α-cholestane-3β,6,8,15,16β,24-hexaol 3-*O*-sulfate
**69**	26.18	$C_{38}H_{63}O_{17}SNa$	823.3791	823.3797	-0.7	[M–Na]⁻	3-*O*-pentosyl-26-*O*-sulfohexosyl-27-nor-5α-ergost-22-ene-3β,6,8,15,26-pentaol
**70**	26.27	$C_{57}H_{93}O_{29}SNa$	1273.5529	1273.5532	-0.2	[M–Na]⁻	dHex–dHex–Glc(-Qui)–Hex–AG V
**71**	27.1	$C_{33}H_{55}O_{14}SNa$	707.3318	707.3323	-0.7	[M–Na]⁻	26-*O*-sulfohexosyl-27-nor-5α-ergost-22-ene-3β,6,8,15,16β,26-hexaol
**72**	27.13	$C_{34}H_{57}O_{14}SNa$	721.3475	721.3464	1.5	[M–Na]⁻	28-*O*-sulfohexosyl-5α-ergost-20(22)-ene-3β,6,8,15,16β,28-hexaol
**73**	27.17	$C_{21}H_{31}O_6SNa$	411.1847	411.1857	-2.4	[M–Na]⁻	3-*O*-sulfoasterone (AG II)
**74**	27.18	$C_{57}H_{91}O_{29}SNa$	1271.5372	1271.5380	-0.6	[M–Na]⁻	dHex–dHex–Qui(-Qui)–DXU–AG VIII
**75**	27.3	$C_{33}H_{57}O_{12}SNa$	677.3576	677.3588	-1.8	[M–Na]⁻	3-*O*-pentosyl-5α-ergostane-3β,6,8,15,26-pentaol 26-*O*-sulfate
**76**	27.4	$C_{39}H_{65}O_{17}SNa$	837.3948	837.3953	-0.6	[M–Na]⁻	3-*O*-pentosyl-28-*O*-sulfohexosyl-5α-ergost-22-ene-3β,6,8,15,28-pentaol
**77**	27.55	$C_{57}H_{93}O_{29}SNa$	1273.5529	1273.5533	-0.3	[M–Na]⁻	dHex–dHex–Qui(-Qui)–DXU–AG VII
**78**	27.67	$C_{62}H_{101}O_{33}SNa$	1405.5951	1405.5958	-0.5	[M–Na]⁻	Hex–dHex–Hex–Xyl(-Qui)–Qui–AG V
**79**	27.81	$C_{56}H_{93}O_{27}SNa$	1229.563	1229.5639	-0.7	[M–Na]⁻	dHex–Pent–Qui(-Qui)–Qui–AG XI
**80**	27.81	$C_{56}H_{91}O_{28}SNa$	1243.5423	1243.5426	-0.2	[M–Na]⁻	dHex–dHex–Glc(-Qui)–Qui–AG X
**81**	27.89	$C_{32}H_{55}O_{12}SNa$	663.342	663.3426	-0.9	[M–Na]⁻	24-*O*-pentosyl-5α-cholestane-3β,6,8,15,24-pentaol 3-*O*-sulfate
**82**	27.98	$C_{33}H_{55}O_{12}SNa$	675.342	675.3424	-0.6	[M–Na]⁻	3-*O*-pentosyl-5α-ergost-20(22)-ene-3β,6,8,15,28-pentaol 28-*O*-sulfate
**83**	28.1	$C_{34}H_{59}O_{14}SNa$	723.3631	723.3638	-1.0	[M–Na]⁻	28-*O*-sulfohexosyl-5α-ergostane-3β,6,8,15,16β,28-hexaol
**84**	28.15	$C_{33}H_{55}O_{13}SNa$	691.3369	691.3378	-1.3	[M–Na]⁻	28-*O*-pentosyl-5α-ergost-22-ene-3β,6,8,15,16β,28-hexaol 3-*O*-sulfate
**85**	28.17	$C_{39}H_{65}O_{17}SNa$	837.3948	837.3958	-1.2	[M–Na]⁻	3-*O*-pentosyl-28-*O*-sulfohexosyl-5α-ergost-22-ene-3β,6,8,15,28-pentaol
**86**	28.38	$C_{39}H_{63}O_{18}SNa$	851.3741	851.3750	-1.1	[M–Na]⁻	Qui–Qui–Qui–AG I
**87**	28.5	$C_{54}H_{87}O_{27}SNa$	1199.5161	1199.5167	-0.5	[M–Na]⁻	dHex–Pent–Xyl(-Qui)–Qui–AG X
**88**	28.5	$C_{63}H_{103}O_{33}SNa$	1419.6108	1419.6115	-0.5	[M–Na]⁻	Hex–dHex–dHex–Glc(-Qui)–Qui–AG V
**89**	28.59	$C_{52}H_{80}NO_{26}SNa$	1166.4695	1166.4709	-1.2	[M–Na]⁻	dHex–dHex–Qui(-Qui)–($C_7H_9NO_4$)–AG II
**90**	28.6	$C_{58}H_{95}O_{30}SNa$	1303.5634	1303.5638	-0.3	[M–Na]⁻	dHex–dHex–Glc(-Qui)–Qui–AG XIV
**91**	28.69	$C_{33}H_{55}O_{14}SNa$	707.3318	707.3324	-0.8	[M–Na]⁻	26-*O*-sulfohexosyl-27-nor-5α-ergost-22-ene-3β,6,8,15,16β,26-hexaol
**92**	28.93	$C_{37}H_{63}O_{16}SNa$	795.3842	795.3849	-0.9	[M–Na]⁻	3-*O*-pentosyl-24-*O*-sulfopentosyl-5α-cholestane-3β,6,8,15,24-pentaol
**93**	29	$C_{57}H_{93}O_{28}SNa$	1257.558	1257.5590	-0.8	[M–Na]⁻	dHex–dHex–Qui(-Qui)–Hex–AG V
**94**	29.21	$C_{39}H_{65}O_{17}SNa$	837.3948	837.3953	-0.6	[M–Na]⁻	3-*O*-pentosyl-26-*O*-sulfohexosyl-5α-ergost-22-ene-3β,6,8,15,26-pentaol
**95**	29.23	$C_{61}H_{99}O_{32}SNa$	1375.5846	1375.5842	0.3	[M–Na]⁻	Hex–dHex–Pent–Xyl(-Qui)–Qui–AG IX
**96**	29.23	$C_{32}H_{53}O_{13}SNa$	677.3212	677.3203	1.3	[M–Na]⁻	24-*O*-pentosyl-5α-cholest-22-ene-3β,6,8,15,16β,24-hexaol 3-*O*-sulfate
**97**	29.25	$C_{56}H_{93}O_{27}SNa$	1229.563	1229.5624	0.5	[M–Na]⁻	dHex–Pent–Xyl(-Qui)–Qui–AG XV
**98**	29.44	$C_{62}H_{103}O_{32}SNa$	1391.6159	1391.6155	0.3	[M–Na]⁻	Hex–dHex–Pent–Xyl(-Qui)–Qui–AG XV
**99**	29.53	$C_{34}H_{57}O_{14}SNa$	721.3475	721.3471	0.6	[M–Na]⁻	28-*O*-sulfohexosyl-5α-ergost-20(22)-ene-3β,6,8,15,16β,28-hexaol
**100**	29.64	$C_{37}H_{63}O_{16}SNa$	795.3842	795.3835	0.9	[M–Na]⁻	3-*O*-pentosyl-24-*O*-sulfopentosyl-5α-cholestane-3β,6,8,15,24-pentaol
**101**	29.7	$C_{27}H_{47}O_{10}SNa$	563.2895	563.2901	-1.1	[M–Na]⁻	5α-cholestane-3β,6,7,8,15α,16β,26-heptaol 6-*O*-sulfate
**102**	29.87	$C_{35}H_{59}O_{14}SNa$	735.3631	735.3628	0.4	[M–Na]⁻	29-*O*-sulfohexosyl-5α-stigmast-20(22)-ene-3β,6,8,15,16β,29-hexaol

No[a]	Rt (min)	Elemental composition	Calculated *m/z*	Measured m/z	Δ (ppm)	Ion type	Proposed structures
**103**	29.92	$C_{34}H_{57}O_{13}SNa$	705.3525	705.3527	-0.3	$[M–Na]^-$	28-*O*-sulfohexosyl-5α-ergost-20(22)-ene-3β,6,8,15,28-pentaol
**104**	30.11	$C_{56}H_{93}O_{28}SNa$	1245.558	1245.5576	0.3	$[M–Na]^-$	dHex–Hex–Xyl(-Qui)–Hex–AG XX
**105**	30.3	$C_{58}H_{95}O_{29}SNa$	1287.5685	1287.5681	0.3	$[M–Na]^-$	dHex–dHex–Glc(-Qui)–Hex–AG XIII
**106**	30.3	$C_{62}H_{101}O_{32}SNa$	1389.6002	1389.6003	-0.1	$[M–Na]^-$	dHex–dHex–Hex–Xyl(-Qui)–Qui–AG V
**107**	30.39	$C_{33}H_{57}O_{13}SNa$	693.3525	693.3533	-1.2	$[M–Na]^-$	pycnopodioside C
**108**	30.41	$C_{38}H_{65}O_{16}SNa$	809.3999	809.4004	-0.6	$[M–Na]^-$	3-*O*-pentosyl-24-*O*-methylsulfopentosyl-5α-cholestane-3β,6,8,15,24-pentaol
**109**	30.63	$C_{32}H_{53}O_{12}SNa$	661.3263	661.3266	-0.5	$[M–Na]^-$	24-*O*-pentosyl-5α-cholest-22-ene-3β,6,8,15,24-pentaol 3-*O*-sulfate
**110**	30.67	$C_{62}H_{101}O_{32}SNa$	1389.6002	1389.6007	-0.4	$[M–Na]^-$	Hex–dHex–dHex–Xyl(-Qui)–Qui–AG V
**111**	30.86	$C_{57}H_{93}O_{29}SNa$	1273.5529	1273.5538	-0.7	$[M–Na]^-$	dHex–Hex–Glc(-Qui)–Qui–AG V
**112**	30.99	$C_{62}H_{101}O_{33}SNa$	1405.5951	1405.5959	-0.6	$[M–Na]^-$	Hex–dHex–Hex–Xyl(-Qui)–Hex–AG XVII
**113**	31.05	$C_{34}H_{57}O_{14}SNa$	721.3475	721.3486	-1.5	$[M–Na]^-$	26-*O*-sulfohexosyl-5α-ergost-22-ene-3β,6,8,15,16β,26-hexaol
**114**	31.39	$C_{38}H_{63}O_{16}SNa$	807.3842	807.3844	-0.2	$[M–Na]^-$	3-*O*-pentosyl-28-*O*-sulfopentosyl-5α-ergost-20(22)-ene-3β,6,8,15,28-pentaol
**115**	31.46	$C_{33}H_{55}O_{13}SNa$	691.3369	691.3380	-1.6	$[M–Na]^-$	24-*O*-sulfohexosyl-5α-cholest-20(22)-ene-3β,6,8,15,24-pentaol
**116**	31.71	$C_{63}H_{103}O_{33}SNa$	1419.6108	1419.6112	-0.3	$[M–Na]^-$	Hex–dHex–Hex–Xyl(-Qui)–Qui–AG XIII
**117**	31.72	$C_{27}H_{47}O_{9}SNa$	547.2946	547.2953	-1.3	$[M–Na]^-$	5α-cholestane-3β,6,8,15α,16β,26-hexaol 6-*O*-sulfate
**118**	31.77	$C_{56}H_{91}O_{28}SNa$	1243.5423	1243.5423	0.0	$[M–Na]^-$	Fuc–Gal–Xyl(-Qui)–Qui–AG V (thornasteroside A)
**119**	32.14	$C_{40}H_{67}O_{18}SNa$	867.4054	867.4058	-0.5	$[M–Na]^-$	28-*O*-[sulfohexosyl-hexosyl]-5α-ergost-22-ene-3β,6,8,15,28-pentaol
**120**	32.22	$C_{34}H_{59}O_{14}SNa$	723.3631	723.3636	-0.7	$[M–Na]^-$	28-*O*-sulfohexosyl-5α-ergostane-3β,6,8,15,16β,28-hexaol
**121**	32.27	$C_{57}H_{95}O_{28}SNa$	1259.5736	1259.5748	-1.0	$[M–Na]^-$	dHex–Hex–Qui(-Qui)–Hex–AG XX
**122**	32.31	$C_{57}H_{93}O_{28}SNa$	1257.558	1257.5581	-0.1	$[M–Na]^-$	Fuc–Qui–Glc(-Qui)–Qui–AG V (luidiaquinoside)
**123**	32.36	$C_{62}H_{103}O_{32}SNa$	1391.6159	1391.6166	-0.5	$[M–Na]^-$	Hex–dHex–Hex–Xyl(-Qui)–Qui–AG XX
**124**	32.71	$C_{32}H_{55}O_{13}SNa$	679.3369	679.3380	-1.6	$[M–Na]^-$	24-*O*-sulfopentosyl-5α-cholestane-3β,6,8,15,16β,24-hexaol
**125**	32.88	$C_{33}H_{55}O_{13}SNa$	691.3369	691.3377	-1.2	$[M–Na]^-$	-
**126**	33.07	$C_{34}H_{59}O_{13}SNa$	707.3682	707.3686	-0.6	$[M–Na]^-$	28-*O*-sulfohexosyl-5α-ergostane-3β,6,8,15,28-pentaol
**127**	33.37	$C_{34}H_{57}O_{14}SNa$	721.3475	721.3487	-1.7	$[M–Na]^-$	26-*O*-sulfohexosyl-5α-ergost-22-ene-3β,6,8,15,16β,26-hexaol
**128**	33.47	$C_{56}H_{93}O_{27}SNa$	1229.563	1229.5636	-0.5	$[M–Na]^-$	dHex–Pent–Qui(-Qui)–Hex–AG XX
**129**	33.56	$C_{63}H_{103}O_{32}SNa$	1403.6159	1403.6164	-0.4	$[M–Na]^-$	Hex–dHex–dHex–Qui(-Qui)–Qui–AG V
**130**	33.78	$C_{62}H_{101}O_{32}SNa$	1389.6002	1389.6006	-0.3	$[M–Na]^-$	Hex–dHex–Pent–Xyl(-Qui)–Qui–AG XIII
**131**	33.84	$C_{33}H_{55}O_{13}SNa$	691.3369	691.3371	-0.3	$[M–Na]^-$	26-*O*-sulfohexosyl-27-nor-5α-ergost-20(22)-ene-3β,6,8,15,26-pentaol
**132**	33.85	$C_{55}H_{89}O_{27}SNa$	1213.5317	1213.5320	-0.2	$[M–Na]^-$	dHex–Pent–Xyl(-Qui)–Qui–AG IX
**133**	34.08	$C_{27}H_{47}O_{9}SNa$	547.2946	547.2960	-2.6	$[M–Na]^-$	5α-cholestane-3β,6,8,15β,16β,26-hexaol 6-*O*-sulfate
**134**	34.13	$C_{33}H_{55}O_{12}SNa$	675.342	675.3421	-0.1	$[M–Na]^-$	3-*O*-pentosyl-5α-ergost-22-ene-3β,6,8,15,26-pentaol 26-*O*-sulfate
**135**	34.34	$C_{57}H_{93}O_{28}SNa$	1257.558	1257.5589	-0.7	$[M–Na]^-$	dHex–dHex–Qui(-Qui)–DXU–AG V
**136**	34.36	$C_{27}H_{47}O_{8}SNa$	531.2997	531.3002	-0.9	$[M–Na]^-$	5α-cholestane-3β,6,8,15,24-pentaol 24-*O*-sulfate
**137**	34.41	$C_{61}H_{101}O_{31}SNa$	1361.6053	1361.6050	0.2	$[M–Na]^-$	Hex–dHex–Pent–Xyl(-Qui)–Qui–AG XX
**138**	34.51	$C_{33}H_{55}O_{13}SNa$	691.3369	691.3367	0.3	$[M–Na]^-$	26-*O*-sulfohexosyl-27-nor-5α-ergost-20(22)-ene-3β,6,8,15,26-pentaol
**139**	34.6	$C_{32}H_{55}O_{12}SNa$	663.342	663.3413	1.1	$[M–Na]^-$	coscinasteroside B
**140**	34.72	$C_{57}H_{95}O_{27}SNa$	1243.5787	1243.5788	-0.1	$[M–Na]^-$	dHex–dHex–Qui(-Qui)–Hex–AG XX
**141**	34.8	$C_{27}H_{47}O_{9}SNa$	547.2946	547.2960	-2.6	$[M–Na]^-$	5α-cholestane-3β,6,8,15,16β,24-hexaol 3-*O*-sulfate
**142**	35.03	$C_{32}H_{55}O_{12}SNa$	663.342	663.3426	-0.9	$[M–Na]^-$	-
**143**	35.52	$C_{56}H_{89}O_{27}SNa$	1225.5317	1225.5310	0.6	$[M–Na]^-$	dHex–Pent–Qui(-Qui)–Hex–AG XXI
**144**	35.66	$C_{62}H_{101}O_{31}SNa$	1373.6053	1373.6041	0.9	$[M–Na]^-$	Hex–dHex–dHex–Xyl(-Qui)–Qui–AG XXII
**145**	35.7	$C_{38}H_{65}O_{16}SNa$	809.3999	809.4002	-0.4	$[M–Na]^-$	3-*O*-pentosyl-24-*O*-methylsulfopentosyl-5α-cholestane-3β,6,8,15,24-pentaol
**146**	35.74	$C_{28}H_{47}O_{10}SNa$	575.2895	575.2903	-1.4	$[M–Na]^-$	5α-ergost-22-ene-3β,6,7,8,15α,16β,26-heptaol 6-*O*-sulfate
**147**	36.02	$C_{35}H_{59}O_{14}SNa$	735.3631	735.3623	1.1	$[M–Na]^-$	29-*O*-sulfohexosyl-5α-stigmast-20(22)-ene-3β,6,8,15,16β,29-hexaol
**148**	36.2	$C_{34}H_{57}O_{14}SNa$	721.3475	721.3478	-0.4	$[M–Na]^-$	28-*O*-sulfohexosyl-5α-ergost-22-ene-3β,6,8,15,16β,28-hexaol
**149**	36.33	$C_{62}H_{103}O_{31}SNa$	1375.621	1375.6209	0.1	$[M–Na]^-$	Hex–dHex–dHex–Xyl(-Qui)–Qui–AG XX
**150**	36.87	$C_{57}H_{91}O_{27}SNa$	1239.5474	1239.5470	0.3	$[M–Na]^-$	dHex–dHex–Qui(-Qui)–Hex–AG XXI
**151**	37.13	$C_{33}H_{55}O_{13}SNa$	691.3369	691.3371	-0.3	$[M–Na]^-$	28-*O*-sulfopentosyl-5α-ergost-20(22)-ene-3β,6,8,15,16β,28-hexaol
**152**	37.34	$C_{57}H_{93}O_{28}SNa$	1257.558	1257.5578	0.2	$[M–Na]^-$	dHex–dHex–Glc(-Qui)–Hex–AG XXII
**153**	37.56	$C_{28}H_{47}O_{9}SNa$	559.2946	559.2942	0.7	$[M–Na]^-$	5α-ergost-22-ene-3β,6,8,15,16β,26-hexaol 6-*O*-sulfate
**154**	37.92	$C_{34}H_{57}O_{13}SNa$	705.3525	705.3525	0.0	$[M–Na]^-$	28-*O*-sulfohexosyl-5α-ergost-22-ene-3β,6,8,15,28-pentaol
**155**	37.99	$C_{32}H_{55}O_{12}SNa$	663.342	663.3417	0.5	$[M–Na]^-$	24-*O*-sulfopentosyl-5α-cholestane-3β,6,8,15,24-pentaol
**156**	38.49	$C_{58}H_{92}NO_{28}SNa$	1282.5532	1282.5519	1.0	$[M–Na]^-$	dHex–dHex–Glc(-Qui)–($C_7H_9NO_4$)–AG V
**157**	38.53	$C_{57}H_{93}O_{28}SNa$	1257.558	1257.5571	0.7	$[M–Na]^-$	dHex–Hex–Qui(-Qui)–Hex–AG XXII
**158**	38.58	$C_{64}H_{105}O_{33}SNa$	1433.6264	1433.6251	0.9	$[M–Na]^-$	Hex–dHex–Hex–Xyl(-Qui)–Qui–AG XVI
**159**	38.62	$C_{56}H_{93}O_{27}SNa$	1229.563	1229.5628	0.2	$[M–Na]^-$	Fuc–Gal–Xyl(-Qui)–Qui–AG XVIII (lethasterioside B)

No[a]	Rt (min)	Elemental composition	Calculated *m/z*	Measured m/z	Δ (ppm)	Ion type	Proposed structures
**160**	38.88	$C_{58}H_{95}O_{28}SNa$	1271.5736	1271.5733	0.2	$[M–Na]^-$	dHex–dHex–Glc(-Qui)–Qui–AG XIII
**161**	39.07	$C_{45}H_{73}O_{20}SNa$	965.4421	965.4422	-0.1	$[M–Na]^-$	Qui–Glc–Qui–AG V
**162**	39.19	$C_{35}H_{61}O_{15}SNa$	753.3737	753.3748	-1.5	$[M–Na]^-$	29-*O*-sulfohexosyl-5α-stigmastane-3β,6,7,8,15,16β,29-heptaol
**163**	39.36	$C_{35}H_{61}O_{14}SNa$	737.3788	737.3794	-0.8	$[M–Na]^-$	29-*O*-sulfohexosyl-5α-stigmastane-3β,6,8,15,16β,29-hexaol
**164**	39.45	$C_{34}H_{57}O_{13}SNa$	705.3525	705.3533	-1.1	$[M–Na]^-$	26-*O*-sulfohexosyl-5α-ergost-22-ene-3β,6,8,15,26-pentaol
**165**	39.47	$C_{34}H_{57}O_{14}SNa$	721.3475	721.3481	-0.8	$[M–Na]^-$	28-*O*-sulfohexosyl-5α-ergost-22-ene-3β,6,8,15,16β,28-hexaol
**166**	39.55	$C_{62}H_{101}O_{32}SNa$	1389.6002	1389.6001	0.1	$[M–Na]^-$	Gal–Fuc–Gal–Xyl(-Qui)–Qui–AG XVII (anasteroside A)
**167**	39.55	$C_{35}H_{59}O_{14}SNa$	735.3631	735.3635	-0.5	$[M–Na]^-$	29-*O*-sulfohexosyl-5α-stigmast-20(22)-ene-3β,6,8,15,16β,29-hexaol
**168**	39.92	$C_{57}H_{95}O_{27}SNa$	1243.5787	1243.5785	0.2	$[M–Na]^-$	dHex–dHex–Glc(-Qui)–Qui–AG XX
**169**	40.11	$C_{32}H_{55}O_{12}SNa$	663.342	663.3421	-0.2	$[M–Na]^-$	24-*O*-sulfopentosyl-5α-cholestane-3β,6,8,15,24-pentaol
**170**	40.12	$C_{58}H_{95}O_{28}SNa$	1271.5736	1271.5730	0.5	$[M–Na]^-$	dHex–dHex–Glc(-Qui)–Qui–AG XIII
**171**	40.47	$C_{34}H_{57}O_{13}SNa$	705.3525	705.3534	-1.3	$[M–Na]^-$	28-*O*-sulfohexosyl-5α-ergost-22-ene-3β,6,8,15,28-pentaol
**172**	40.47	$C_{33}H_{57}O_{12}SNa$	677.3576	677.3584	-1.2	$[M–Na]^-$	24-*O*-methylsulfopentosyl-5α-cholestane-3β,6,8,15,24-pentaol
**173**	40.68	$C_{56}H_{91}O_{27}SNa$	1227.5474	1227.5465	0.7	$[M–Na]^-$	dHex–Pent–Qui(-Qui)–Hex–AG XXII
**174**	40.8	$C_{27}H_{41}O_{8}SNa$	525.2528	525.2523	1.0	$[M–Na]^-$	AG VIII
**175**	40.87	$C_{57}H_{93}O_{27}SNa$	1241.563	1241.5623	0.6	$[M–Na]^-$	dHex–dHex–Qui(-Qui)–Qui–AG V
**176**	40.87	$C_{63}H_{103}O_{32}SNa$	1403.6159	1403.6157	0.1	$[M–Na]^-$	Hex–dHex–dHex–Glc(-Qui)–Qui–AG XXII
**177**	41.41	$C_{55}H_{91}O_{26}SNa$	1199.5525	1199.5522	0.3	$[M–Na]^-$	dHex–Pent–Xyl(-Qui)–Qui–AG XVIII
**178**	41.8	$C_{27}H_{43}O_{8}SNa$	527.2684	527.2682	0.4	$[M–Na]^-$	AG VII
**179**	41.98	$C_{34}H_{57}O_{13}SNa$	705.3525	705.3539	-2.0	$[M–Na]^-$	26-*O*-sulfohexosyl-5α-ergost-22-ene-3β,6,8,15,26-pentaol
**180**	42.17	$C_{57}H_{89}O_{26}SNa$	1221.5368	1221.5369	-0.1	$[M–Na]^-$	dHex–dHex–Qui(-Qui)–($C_6H_6O_3$)–AG V
**181**	42.2	$C_{35}H_{61}O_{14}SNa$	737.3788	737.3792	-0.5	$[M–Na]^-$	29-*O*-sulfohexosyl-5α-stigmastane-3β,6,8,15,16β,29-hexaol
**182**	42.31	$C_{57}H_{93}O_{27}SNa$	1241.563	1241.5628	0.2	$[M–Na]^-$	dHex–dHex–Qui(-Qui)–Hex–AG XXII
**183**	42.4	$C_{35}H_{59}O_{14}SNa$	735.3631	735.3632	-0.1	$[M–Na]^-$	29-*O*-sulfohexosyl-5α-stigmast-22-ene-3β,6,8,15,16β,29-hexaol
**184**	42.55	$C_{33}H_{55}O_{12}SNa$	675.342	675.3424	-0.6	$[M–Na]^-$	28-*O*-sulfopentosyl-5α-ergost-20(22)-ene-3β,6,8,15,28-pentaol
**185**	42.96	$C_{35}H_{61}O_{13}SNa$	721.3838	721.3845	-1.0	$[M–Na]^-$	29-*O*-sulfohexosyl-5α-stigmastane-3β,6,8,15,29-pentaol
**186**	43.07	$C_{58}H_{92}NO_{27}SNa$	1266.5583	1266.5584	-0.1	$[M–Na]^-$	dHex–dHex–Qui(-Qui)–($C_7H_9NO_4$)–AG V
**187**	43.19	$C_{57}H_{89}O_{26}SNa$	1221.5368	1221.5374	-0.5	$[M–Na]^-$	dHex–dHex–Qui(-Qui)–($C_6H_6O_3$)–AG V
**188**	43.69	$C_{33}H_{55}O_{12}SNa$	675.342	675.3427	-1.0	$[M–Na]^-$	28-*O*-sulfopentosyl-5α-ergost-20(22)-ene-3β,6,8,15,28-pentaol
**189**	43.86	$C_{35}H_{59}O_{14}SNa$	735.3631	735.3640	-1.2	$[M–Na]^-$	29-*O*-sulfohexosyl-5α-stigmast-22-ene-3β,6,8,15,16β,29-hexaol
**190**	43.97	$C_{57}H_{95}O_{27}SNa$	1243.5787	1243.5799	-1.0	$[M–Na]^-$	dHex–dHex–Qui(-Qui)–DXU–AG XVIII
**191**	44.75	$C_{58}H_{95}O_{28}SNa$	1271.5436	1271.5448	-0.9	$[M–Na]^-$	dHex–dHex–Qui(-Qui)–DXU–AG XIII
**192**	44.81	$C_{62}H_{101}O_{31}SNa$	1373.6053	1373.6056	-0.2	$[M–Na]^-$	dHex–dHex–Hex–Xyl(-Qui)–Qui–AG XXII
**193**	45.81	$C_{62}H_{101}O_{31}SNa$	1373.6053	1373.6053	0.0	$[M–Na]^-$	Hex–dHex–dHex–Xyl(-Qui)–Qui–AG XVII
**194**	45.82	$C_{63}H_{103}O_{32}SNa$	1403.6159	1403.6164	-0.4	$[M–Na]^-$	Hex–dHex–Hex–Xyl(-Qui)–Qui–AG XXIII
**195**	45.99	$C_{57}H_{91}O_{27}SNa$	1239.5474	1239.5473	0.1	$[M–Na]^-$	dHex–dHex–Qui(-Qui)–DXU–AG XIX
**196**	46.45	$C_{32}H_{53}O_{12}SNa$	661.3263	661.3261	0.3	$[M–Na]^-$	24-*O*-sulfopentosyl-5α-cholest-22-ene-3β,6,8,15,24-pentaol
**197**	47.6	$C_{35}H_{61}O_{14}SNa$	737.3788	737.3793	-0.7	$[M–Na]^-$	29-*O*-sulfohexosyl-5α-stigmastane-3β,6,8,15,16β,29-hexaol
**198**	48.4	$C_{56}H_{91}O_{27}SNa$	1227.5474	1227.5470	0.3	$[M–Na]^-$	Fuc–Gal–Xyl(-Qui)–Qui–AG XVII (lethasterioside A)
**199**	48.83	$C_{58}H_{92}NO_{27}SNa$	1266.5583	1266.5580	0.2	$[M–Na]^-$	dHex–dHex–Qui(-Qui)–($C_7H_9NO_4$)–AG V
**200**	50.2	$C_{57}H_{93}O_{27}SNa$	1241.563	1241.5635	-0.4	$[M–Na]^-$	dHex–dHex–Glc(-Qui)–Qui–AG XVII
**201**	50.98	$C_{63}H_{103}O_{31}SNa$	1387.621	1387.6211	-0.1	$[M–Na]^-$	Hex–dHex–dHex–Qui(-Qui)–Qui–AG XVII
**202**	52.5	$C_{33}H_{57}O_{12}SNa$	677.3576	677.3588	-1.8	$[M–Na]^-$	24-*O*-methylsulfopentosyl-5α-cholestane-3β,6,8,15,24-pentaol
**203**	53.28	$C_{57}H_{93}O_{27}SNa$	1241.563	1241.5636	-0.5	$[M–Na]^-$	dHex–dHex–Qui(-Qui)–DXU–AG XVII
**204**	55.29	$C_{34}H_{59}O_{13}SNa$	707.3682	707.3689	-1.0	$[M–Na]^-$	29-*O*-sulfopentosyl-5α-stigmastane-3β,6,8,15,16β,29-hexaol
**205**	59.82	$C_{27}H_{43}O_{7}SNa$	511.2735	511.2749	-2.7	$[M–Na]^-$	3-*O*-sulfothornasterol A (AG V)
**206**	66.76	$C_{57}H_{93}O_{26}SNa$	1225.5681	1225.5685	-0.3	$[M–Na]^-$	dHex–dHex–Qui(-Qui)–Qui–AG XVII
**207**	72.27	$C_{27}H_{43}O_{6}SNa$	495.2786	495.2795	-1.8	$[M–Na]^-$	3-O-sulfo-24,25-dihydromarthasterone (AG XVII)

[a] The number of the peaks on (–)LC/MS chromatogram

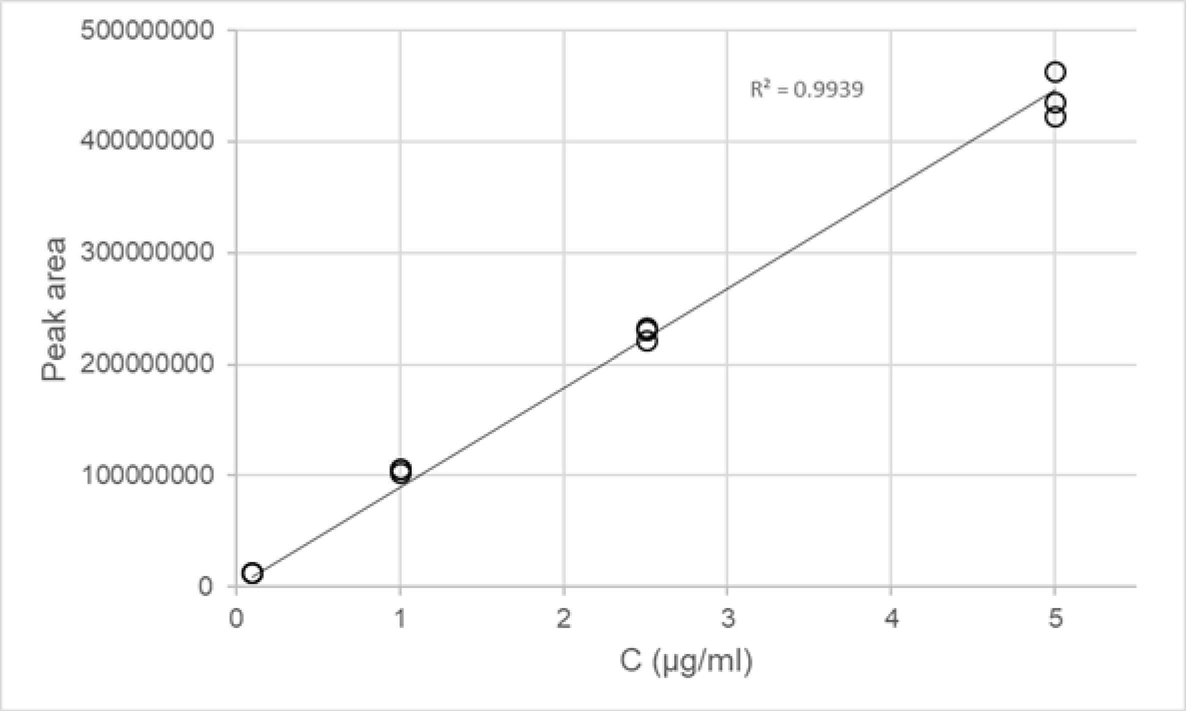

Figure 2S. Calibration curve for fuscaside A (ion [M – Na]⁻ at *m/z* 795.38)

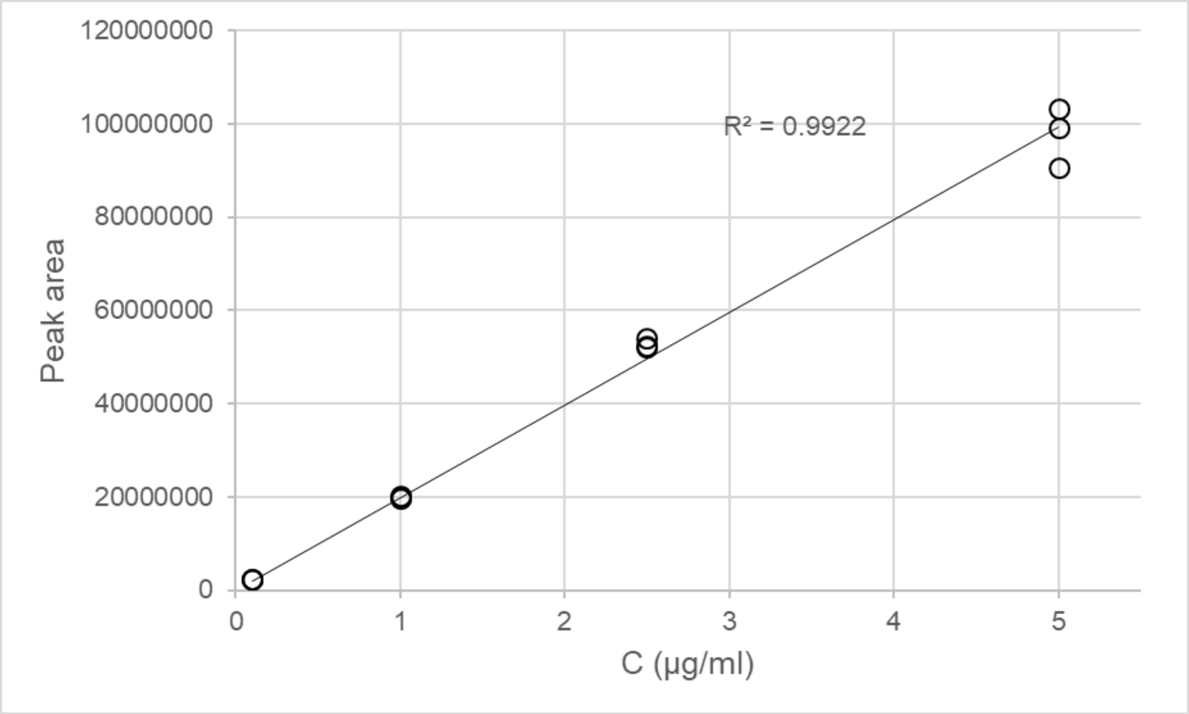

Figure 3S. Calibration curve for lethasterioside A (ion [M – Na]⁻ at *m/z* 1227.54)

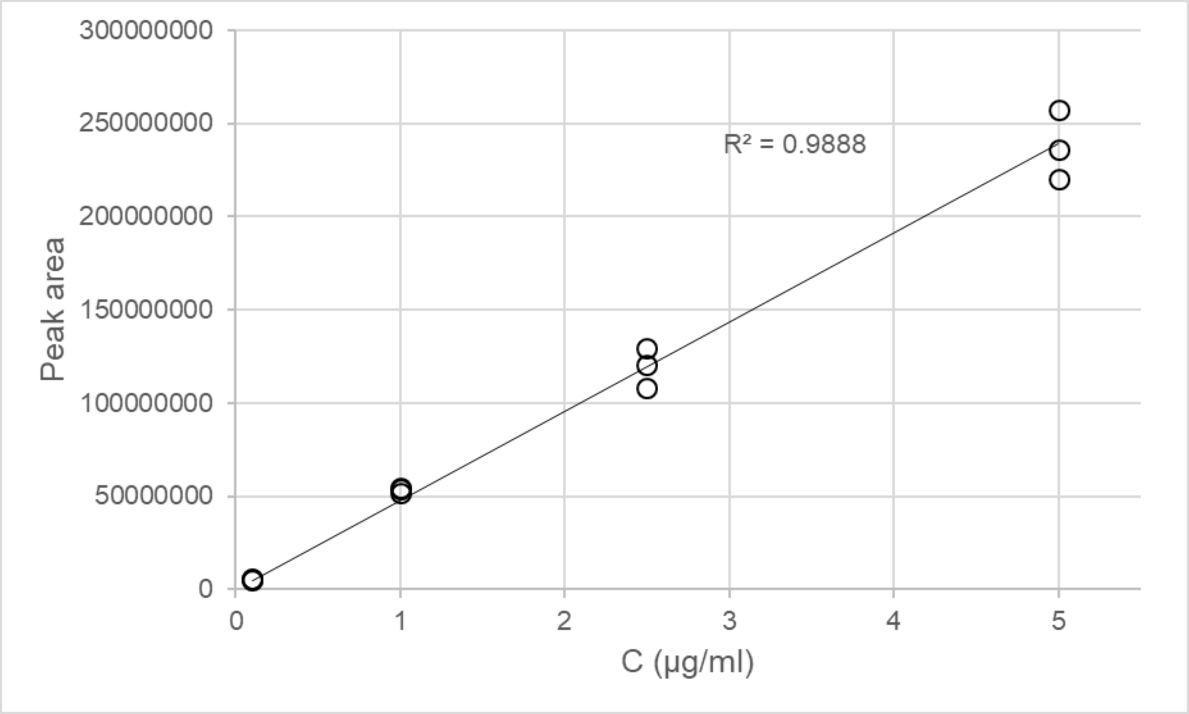

Figure 4S. Calibration curve for 5α-cholestan-3β,4β,6α,7α,8,15β,16β,26-octaol (for the sum of ion intensities $[M-H]^-$ at *m*/*z* 499.33 and $[M+FA]^-$ at *m*/*z* 545.3)

Table S4 Content of detected compounds in different organs and coelomic fluid of the starfish *L. fusca* (ng/g wet weight of the organs for BW, G, PC, and S and ng/ml for CF) and result of statistical analysis (ANOVA followed by Tukey HSD test of multiple comparisons was performed for BW, G, PC, and S groups; q-value < 0.05 was considered statistically significant).

	Body Walls					Coelomic fluid					Gonads				Pyloric caeca					Stomach					*p-value*	*q-value*	Tukey's HSD
N	BW#1	BW#2	BW#3	BW#4	BW#5	CF1	CF2	CF3	CF4	CF5	G#1	G#2	G#4	G#5	PC#1	PC#2	PC#3	PC#4	PC#5	S#1	S#2	S#3	S#4	S#5			
**1**	0.1	1.0	1.3	2.1	0.9	nd	nd	nd	nd	nd	0.5	<0.1	1.6	15.2	152.9	175.5	334.7	757.5	428.4	3.8	2.1	20.2	14.0	6.9	0.001	0.003	PC-BW; PC-G; S-PC
**2**	0.4	0.2	0.9	3.9	2.2	nd	nd	nd	nd	nd	0.2	nd	2.7	11.1	167.7	171.0	375.4	952.5	441.0	8.4	3.3	12.5	22.7	9.3	0.002	0.009	PC-BW; PC-G; S-PC
**3**	1.1	55.1	40.1	36.2	4.8	0.1	0.1	nd	nd	nd	nd	0.8	1.9	0.3	0.1	0.2	0.2	2.3	1.4	5.3	6.6	7.0	7.9	4.1	0.011	0.034	G-BW; PC-BW; S-BW
**4**	0.2	0.1	0.1	0.3	0.1	nd	nd	nd	nd	nd	nd	nd	nd	1.0	122.8	52.8	132.6	202.4	108.4	1.3	0.6	1.2	1.5	0.8	<0.001	<0.001	PC-BW; PC-G; S-PC
**5**	28.4	11.6	4.3	23.4	4.5	nd	0.1	nd	nd	nd	54.0	38.7	26.1	33.1	3304.0	12328.5	681.4	3065.1	262.5	56.7	101.7	19.2	64.2	7.5	0.068	0.105	-
**6**	242.4	1244.9	2.4	17.9	1059.9	0.6	2.5	nd	nd	1.8	26.0	37.8	4.3	57.8	32.2	31.0	33.9	86.5	70.2	15.8	24.2	39.0	46.2	33.8	0.068	0.133	-
**7**	0.3	0.7	2.9	21.6	168.2	nd	nd	nd	nd	nd	3.7	1.3	9.8	307.3	372.0	239.2	1207.3	3870.7	13159.6	6.4	4.7	28.4	69.0	84.0	0.137	0.161	-
**8**	0.1	nd	0.2	0.3	nd	nd	nd	nd	nd	nd	61.5	3.3	2.0	1.7	2.2	1.5	1.6	6.3	2.6	32.6	26.4	45.7	172.0	32.3	0.046	0.099	-
**9**	85.1	441.5	3.3	6.7	305.6	0.3	1.3	0.1	<0.1	0.7	23.4	23.2	2.8	23.7	13.6	11.4	20.5	49.8	32.7	28.4	36.1	43.0	97.7	47.6	0.126	0.198	-
**10**	29.8	449.3	0.4	3.1	4.2	<0.1	0.3	<0.1	<0.1	nd	34.7	21.4	3.7	14.9	5697.7	21395.2	687.9	3043.8	441.0	84.2	149.3	8.2	39.0	9.0	0.117	0.145	-
**11**	0.6	0.2	0.1	0.5	0.6	nd	nd	nd	nd	nd	0.1	<0.1	0.4	3.6	340.6	89.4	171.3	752.5	275.9	5.6	2.2	7.2	19.0	7.2	0.003	0.01	PC-BW; PC-G; S-PC
**12**	0.4	0.4	3.1	9.5	78.3	nd	nd	nd	nd	nd	3.0	0.8	7.3	323.1	391.1	238.7	428.9	4106.0	13195.0	1.2	2.1	18.3	38.2	124.9	0.164	0.186	-
**13**	2.2	335.1	0.8	0.6	78.4	nd	1.0	nd	nd	nd	15.3	17.4	nd	5.0	9.0	17.0	10.8	28.7	19.8	11.6	11.5	10.4	6.0	5.2	0.369	0.426	-
**14**	2.2	12.0	<0.1	<0.1	nd	nd	nd	nd	nd	nd	1.9	1.2	nd	0.1	502.1	1299.9	17.0	38.9	20.0	6.2	9.0	nd	2.6	0.7	0.147	0.171	-
**15**	2.4	0.9	1.3	4.4	2.1	nd	nd	nd	nd	nd	1.4	0.7	4.4	24.6	855.5	734.2	1036.8	1989.9	467.7	14.6	5.3	20.7	28.8	7.0	<0.001	0.001	PC-BW; PC-G; S-PC
**16**	nd	0.1	nd	nd	0.4	nd	nd	nd	nd	nd	nd	nd	nd	nd	0.6	1.0	3.0	9.2	5.2	85.5	156.7	nd	29.2	167.1	0.006	0.024	S-BW; S-G; S-PC
**17**	22.2	84.9	14.0	3.0	126.4	nd	nd	nd	nd	nd	70.2	34.7	12.0	42.0	95.3	143.2	7.8	21.7	128.4	442.5	670.4	425.3	777.0	975.2	<0.001	<0.001	S-BW; S-G; S-PC
**18**	3.4	13.4	13.8	39.8	44.2	nd	nd	nd	nd	nd	9.0	4.4	36.7	232.3	752.0	1594.4	2317.6	7797.0	3920.7	11.5	78.7	185.8	387.0	174.8	0.007	0.018	PC-BW; PC-G; S-PC
**19**	0.1	nd	0.3	0.2	<0.1	nd	nd	nd	nd	nd	nd	nd	nd	0.2	32.0	3.9	294.4	95.7	12.6	2.4	1.4	35.0	5.8	1.3	0.127	0.15	-
**20**	25.8	159.8	0.8	0.2	26.9	nd	0.7	nd	nd	nd	93.3	12.5	nd	2.8	27.4	10.7	5.0	12.6	7.1	202.2	25.9	8.1	12.7	10.2	0.738	0.738	-
**21**	3.2	21.2	19.0	25.3	20.1	nd	nd	nd	nd	nd	11.3	7.1	28.1	117.8	1226.7	1934.7	2332.9	4028.9	1244.5	16.0	78.9	109.9	152.2	44.4	<0.001	0.001	PC-BW; PC-G; S-PC
**22**	55.2	79.0	45.4	2.9	104.6	nd	nd	nd	nd	nd	2283.3	295.6	56.8	132.5	401.7	294.5	33.7	64.5	574.2	1810.9	302.4	346.7	554.8	724.3	0.243	0.316	-
**23**	24.4	46.0	0.3	0.3	60.3	<0.1	nd	nd	nd	nd	16.6	5.4	3.3	304.2	2056.6	1794.4	63.9	247.1	3736.0	79.4	46.6	9.9	20.5	145.8	0.016	0.036	PC-BW; PC-G; S-PC
**24**	0.1	nd	0.3	8.7	0.1	nd	nd	nd	nd	nd	1.6	nd	9.6	8.1	23.7	4.5	161.9	1924.3	1.4	3.0	1.5	52.2	277.4	0.8	0.408	0.413	-
**25**	0.5	0.8	0.5	1.5	0.4	nd	nd	nd	nd	nd	24.9	6.4	8.5	19.5	5.1	8.3	6.1	21.0	7.8	609.4	801.7	229.4	3538.2	460.4	0.058	0.119	-
**26**	60.9	2463.1	535.4	5561.0	98.7	4.5	15.2	0.9	7.1	1.0	342.8	157.4	1453.2	140.3	75.4	62.7	41.6	207.8	52.6	134.0	76.9	821.2	279.2	67.3	0.199	0.269	-
**27**	96.0	15.1	351.7	69.9	204.4	0.1	nd	nd	1.0	nd	962.8	208.3	574.3	179.6	117.7	185.8	120.0	196.1	228.5	1569.7	2859.7	18224.1	27866.8	4666.2	0.028	0.07	-
**28**	28.0	1787.1	3301.8	4144.6	120.0	3.2	11.4	2.4	3.7	0.1	25.4	131.3	985.5	92.8	19.7	46.2	42.4	125.4	39.7	322.5	323.2	1743.4	372.7	142.1	0.063	0.126	-
**29**	24.0	30.6	0.9	0.1	12.4	nd	nd	nd	nd	nd	1555.7	40.7	13.2	16.7	91.5	5.2	7.7	15.5	11.7	634.5	38.2	98.8	47.4	33.7	0.394	0.442	-
**30**	0.2	164.2	50.0	53.6	2.3	0.2	0.9	0.3	0.1	<0.1	9.6	7.4	12.9	6.1	14.4	21.2	52.6	122.9	74.6	15.6	405.3	683.9	1215.8	24.4	0.051	0.085	-
**31**	1.0	50.4	9.5	18.2	9.1	nd	nd	nd	nd	nd	104.4	10.2	30.5	33.9	21.2	18.0	17.5	53.0	60.5	818.5	685.0	2984.7	8560.5	1376.3	0.044	0.099	-
**32**	0.2	1.1	2.0	0.7	0.1	nd	nd	nd	nd	nd	4.4	2.2	0.7	6.6	5.2	7.0	3.7	12.6	8.4	1247.7	931.4	44.4	1379.4	487.3	0.001	0.004	S-BW; S-G; S-PC

	Body Walls					Coelomic fluid					Gonads				Pyloric caeca					Stomach					*p-value*	*q-value*	Tukey's HSD
N	BW#1	BW#2	BW#3	BW#4	BW#5	CF1	CF2	CF3	CF4	CF5	G#1	G#2	G#4	G#5	PC#1	PC#2	PC#3	PC#4	PC#5	S#1	S#2	S#3	S#4	S#5			
**33**	161.7	22.0	13.2	1.3	29.0	nd	nd	nd	0.4	nd	1085.2	41.3	40.1	16.0	50.8	17.2	5.8	14.3	13.5	2298.9	1451.8	1744.3	5812.5	2437.7	0.001	0.005	S-BW; S-G; S-PC
**34**	25.8	83.3	144.2	161.8	51.0	0.1	0.3	0.1	<0.1	0.2	18.7	3.0	11.0	9.0	20.1	6.2	9.8	21.5	11.6	96.2	23.2	68.3	31.0	23.7	0.008	0.026	G-BW; PC-BW
**35**	39.0	10.0	8.5	1.9	11.8	nd	nd	nd	nd	nd	1838.9	21.3	20.7	6.6	62.2	7.7	4.2	9.1	8.2	3387.0	235.0	341.7	793.1	338.6	0.199	0.269	-
**36**	28.3	10.5	9.6	2.3	12.0	nd	nd	nd	nd	nd	1083.7	18.1	12.9	5.9	31.5	6.3	4.7	11.9	8.7	951.2	165.3	249.4	571.6	244.7	0.098	0.171	-
**37**	53.5	2.1	1.0	0.2	1.8	nd	nd	nd	nd	nd	363.1	23.2	2.6	0.7	279.0	7.8	4.9	12.1	6.4	2880.2	105.3	3.1	28.0	31.9	0.477	0.509	-
**38**	0.2	32.5	389.3	143.4	1.9	<0.1	nd	nd	<0.1	nd	356.8	6.2	202.1	27.7	28.7	13.8	305.2	681.5	82.2	179.5	68.7	21617.9	18196.8	110.2	0.109	0.177	-
**39**	2.0	362.1	727.6	1029.4	3.3	0.3	1.7	0.1	0.7	nd	36.3	28.0	129.9	15.9	41.3	29.4	38.0	84.0	24.1	457.5	96.3	415.0	107.1	72.2	0.109	0.177	-
**40**	15.8	26.6	7.6	3.2	66.1	nd	nd	nd	nd	nd	5.8	4.5	0.4	40.7	17.4	22.8	1.1	7.5	42.8	766.2	920.0	34.8	393.6	962.7	0.001	0.004	S-BW; S-G; S-PC
**41**	0.4	1.3	0.4	0.6	1.7	nd	nd	nd	nd	nd	1.3	1.5	nd	20.1	4.1	6.9	0.3	1.8	15.9	299.0	513.3	16.6	286.2	511.3	<0.001	0.003	S-BW; S-G; S-PC
**42**	9.5	1334.4	29.4	88.8	13.1	2.5	5.1	2.2	1.6	2.3	126.9	58.4	24.8	23.7	76.3	42.1	45.6	156.6	47.6	352.1	442.9	6963.1	6687.8	162.4	0.081	0.151	-
**43**	409.1	8.7	0.2	0.4	4.0	<0.1	nd	nd	0.6	1.0	18306.4	92.9	110.7	13.0	1073.3	17.0	6.3	17.1	11.6	13986.7	265.0	71.2	85.0	137.2	0.509	0.53	-
**44**	42.9	69.0	24.8	2.1	183.4	nd	0.1	nd	nd	0.1	18.3	3.2	0.5	13.8	6.7	5.9	0.5	3.5	11.2	730.2	310.0	161.4	136.4	412.9	0.002	0.01	S-BW; S-G; S-PC
**45**	48.5	14.8	0.1	1.8	0.9	nd	nd	nd	nd	nd	12.4	1.3	1.6	9.4	2787.4	477.6	34.8	94.5	92.2	137.4	8.5	2.1	8.4	7.7	0.25	0.277	-
**46**	13.5	80.0	0.2	4.3	19.9	nd	<0.1	nd	nd	nd	12.7	5.3	3.0	40.2	743.7	2117.0	60.9	243.7	344.9	57.7	51.3	6.3	20.6	17.1	0.06	0.094	-
**47**	16.0	1.3	0.8	1.1	1.8	nd	nd	nd	nd	nd	91.6	5.0	2.3	2.4	98.8	4.5	3.7	10.8	7.8	952.6	17.4	28.1	41.5	30.3	0.407	0.451	-
**48**	3.7	2.0	2.8	2.4	199.6	0.3	0.1	<0.1	<0.1	0.1	9.2	1.8	5.9	718.4	295.4	75.4	107.8	207.4	7882.2	30.4	7.6	26.0	17.1	473.0	0.423	0.423	-
**49**	29.9	156.4	88.4	2.0	153.8	nd	0.2	nd	nd	nd	36.9	8.4	1.6	8.6	8.0	8.8	4.5	4.2	4.7	203.2	199.9	805.2	126.7	78.7	0.052	0.108	-
**50**	100.1	6.3	3.5	0.9	3.8	nd	nd	nd	<0.1	nd	139.5	29.0	72.2	12.4	452.3	30.5	9.8	32.8	36.7	140.6	145.5	367.3	660.0	381.7	0.023	0.06	-
**51**	5.8	385.9	1.0	31.9	5.7	nd	0.1	nd	nd	nd	55.2	32.1	4.9	13.3	51.4	66.9	25.3	38.6	45.0	843.1	2228.3	291.3	1226.2	709.9	0.002	0.009	S-BW; S-G; S-PC
**52**	82.0	14.7	8.3	1.1	23.7	nd	nd	nd	0.1	nd	7071.3	26.7	44.3	5.1	42.1	4.1	4.3	14.2	8.1	6736.8	57.9	87.0	102.4	107.8	0.505	0.53	-
**53**	1.9	3.4	2.4	45.1	0.3	nd	nd	nd	nd	nd	2.1	0.6	17.4	13.3	139.2	92.1	253.5	299.3	39.7	11.7	4.6	13.4	103.7	3.5	0.003	0.01	PC-BW; PC-G; S-PC
**54**	21.7	114.3	34.3	140.5	10.1	0.3	0.7	0.1	0.1	nd	7473.3	80.6	268.9	45.8	507.0	30.2	133.8	396.3	54.9	835.4	72.8	3892.5	1345.9	260.7	0.383	0.434	-
**55**	13.7	35.5	24.9	146.9	20.5	0.3	0.2	0.2	0.1	0.1	5.3	7.4	64.6	80.0	829.8	110.1	3241.0	8552.8	196.7	13.6	13.2	141.4	418.6	14.8	0.121	0.147	-
**56**	2.8	3.0	8.7	39.6	0.1	nd	nd	nd	nd	nd	2.5	1.1	16.4	10.9	227.0	219.1	502.4	2626.5	22.2	13.1	10.5	20.1	95.6	2.8	0.165	0.186	-
**57**	1.4	30.2	0.6	0.2	4.8	nd	nd	nd	nd	nd	3.4	2.2	0.6	10.2	158.9	767.9	49.7	63.8	123.3	6.1	13.9	2.9	5.3	4.1	0.092	0.124	-
**58**	43.1	39.9	105.7	103.8	31.6	<0.1	nd	<0.1	<0.1	nd	15.1	8.3	47.8	144.2	2348.6	1420.6	4231.9	5110.9	1115.1	89.0	64.4	160.4	251.9	61.4	<0.001	0.002	PC-BW; PC-G; S-PC
**59**	34.1	142.0	9.6	33.2	30.0	0.5	0.7	0.3	0.2	0.3	28.7	9.2	19.6	97.6	2323.1	5039.5	414.5	1843.3	1027.4	122.8	175.6	27.2	136.1	47.2	0.006	0.018	PC-BW; PC-G; S-PC
**60**	44.0	70.7	28.5	2.0	258.2	<0.1	0.1	nd	nd	nd	93.2	28.9	9.1	76.8	74.3	49.1	18.0	29.0	166.1	1841.5	1075.2	620.6	657.9	1964.5	<0.001	0.001	S-BW; S-G; S-PC
**61**	0.7	0.1	0.6	nd	0.5	nd	nd	nd	nd	nd	215.6	3.0	2.8	0.7	14.9	1.0	2.4	9.0	3.1	142.7	37.4	58.6	401.9	142.9	0.049	0.104	-
**62**	32.0	1.2	0.3	0.1	1.8	nd	nd	nd	nd	nd	1483.7	5.9	11.3	3.4	117.8	1.6	7.5	57.9	7.0	943.7	5.0	8.8	4.1	10.1	0.511	0.53	-
**63**	9.4	23.6	0.5	0.6	26.9	<0.1	nd	0.1	nd	<0.1	5.2	1.5	0.9	60.0	530.5	829.1	60.6	41.4	815.8	19.6	43.1	8.3	12.9	30.1	0.008	0.019	PC-BW; PC-G; S-PC
**64**	62.7	116.2	472.1	2212.9	610.7	1.0	1.1	1.1	0.7	1.2	74.6	28.6	988.5	2157.7	2987.5	2438.9	17721.8	64535.2	15898.9	261.3	152.3	1204.6	3448.2	612.0	0.078	0.115	-
**65**	0.6	0.9	<0.1	0.4	0.3	nd	nd	nd	nd	nd	0.2	0.4	nd	4.3	1.6	2.3	2.0	7.7	6.1	260.8	859.3	1.7	94.0	660.6	0.017	0.046	S-BW; S-G; S-PC
**66**	22.4	51.4	37.3	8.2	65.4	0.1	<0.1	nd	0.6	nd	16.8	37.4	22.9	72.2	36.0	69.6	50.5	132.7	124.7	2048.8	1763.3	465.0	15822.2	1742.5	0.147	0.22	-
**67**	47.4	443.0	61.9	10.0	449.9	0.5	1.5	nd	nd	nd	4.6	10.5	0.1	15.4	3.1	7.8	0.3	2.1	7.6	82.7	355.3	40.5	71.3	73.6	0.1	0.172	-
**68**	41.4	113.9	7.8	16.0	1.2	<0.1	0.1	nd	<0.1	nd	20.8	7.4	4.0	8.2	2263.3	3657.0	238.2	384.4	69.6	155.8	58.1	10.3	24.7	6.8	0.06	0.094	-

	Body Walls					Coelomic fluid					Gonads				Pyloric caeca					Stomach					*p-value*	*q-value*	Tukey's HSD
N	BW#1	BW#2	BW#3	BW#4	BW#5	CF1	CF2	CF3	CF4	CF5	G#1	G#2	G#4	G#5	PC#1	PC#2	PC#3	PC#4	PC#5	S#1	S#2	S#3	S#4	S#5			
**69**	12.3	78.1	1.5	9.0	11.4	nd	0.1	nd	nd	nd	12.0	6.0	4.8	32.4	684.6	2114.1	132.2	459.5	353.5	42.7	38.9	7.6	22.1	10.3	0.03	0.058	-
**70**	38.7	43.1	35.8	8.5	48.0	0.4	0.2	nd	0.2	1.0	55.3	35.7	14.8	51.8	14.5	23.9	13.4	26.6	41.4	1897.0	2821.0	1854.2	3607.9	3845.4	<0.001	<0.001	S-BW; S-G; S-PC
**71**	17.3	16.5	9.1	10.3	0.1	<0.1	nd	nd	nd	nd	5.7	2.0	5.0	5.2	1055.4	482.8	387.7	596.2	24.4	44.1	13.9	13.7	24.2	2.9	0.002	0.007	PC-BW; PC-G; S-PC
**72**	33.1	15.3	5.9	69.8	2.5	<0.1	0.1	<0.1	nd	nd	11.4	4.0	35.4	25.5	2031.1	599.4	360.6	4368.5	92.6	98.3	22.3	19.2	180.9	8.2	0.06	0.094	-
**73**	167.6	100.7	78.6	30.3	172.6	0.8	0.8	0.3	0.6	0.9	2986.5	147.1	135.1	154.8	1276.5	430.1	194.4	673.2	382.3	2266.6	614.8	1669.2	2807.9	1149.3	0.045	0.099	-
**74**	1.6	8.5	1.2	5.7	7.2	0.4	0.4	nd	<0.1	1.4	18.4	6.5	6.5	16.7	8.1	7.8	7.7	18.4	16.2	1490.5	1492.8	215.2	2879.9	1265.2	<0.001	0.004	S-BW; S-G; S-PC
**75**	0.5	5.3	37.0	39.3	0.4	nd	nd	nd	nd	nd	2.0	2.6	21.4	14.3	51.0	184.4	1946.5	2297.2	27.3	4.2	13.7	107.0	116.0	2.2	0.076	0.115	-
**76**	0.8	1.8	16.9	10.0	1.5	nd	nd	nd	nd	nd	0.4	1.1	5.1	6.4	37.1	11.4	963.1	595.9	44.8	2.2	4.1	32.0	19.9	2.2	0.091	0.124	-
**77**	11.2	17.2	8.4	9.1	19.2	0.1	0.1	nd	nd	0.1	66.2	12.2	11.8	32.2	23.6	21.0	11.0	32.8	28.1	4120.9	3021.4	302.2	4763.9	2234.7	<0.001	0.002	S-BW; S-G; S-PC
**78**	1247.1	4523.6	960.5	226.2	5384.2	5.6	9.3	1.2	5.9	7.7	2984.7	1785.1	372.0	2382.7	1179.5	1300.9	71.3	121.9	2092.6	25461.0	22508.6	17412.0	16285.3	35490.6	<0.001	<0.001	S-BW; S-G; S-PC
**79**	28.3	7.3	5.8	1.1	9.0	nd	nd	nd	nd	nd	682.6	4.2	6.7	0.2	68.5	1.5	3.8	9.4	4.7	863.5	5.2	13.2	7.8	11.0	0.579	0.584	-
**80**	32.3	21.9	114.0	0.9	37.4	nd	nd	nd	nd	0.1	198.1	25.2	11.4	19.2	28.1	15.7	34.4	16.8	19.3	401.3	407.8	4922.6	829.8	231.1	0.159	0.231	-
**81**	192.7	222.8	33.2	83.3	356.9	0.8	1.0	0.6	0.5	0.6	76.0	21.0	49.7	1205.1	10565.5	7590.5	1793.2	5272.2	10762.4	597.2	137.5	77.5	190.2	547.2	<0.001	0.001	PC-BW; PC-G; S-PC
**82**	0.5	4.1	163.8	147.5	0.7	1.3	1.2	1.5	1.1	1.5	9.9	10.7	83.4	50.7	100.1	165.6	7589.5	7869.6	104.2	13.5	36.2	476.4	456.4	9.7	0.101	0.132	-
**83**	19.6	46.4	1.7	21.2	1.8	nd	<0.1	nd	nd	nd	9.9	4.6	11.2	9.8	1093.5	1578.9	123.0	1371.5	20.0	57.5	48.6	6.8	61.0	3.7	0.007	0.019	PC-BW; PC-G; S-PC
**84**	24.3	24.9	152.2	96.1	9.8	0.1	<0.1	0.2	<0.1	nd	4.2	5.9	45.4	30.5	113.1	116.3	6812.0	5248.2	82.8	12.3	27.3	201.5	182.6	6.7	0.101	0.132	-
**85**	14.9	88.6	1.0	7.3	11.7	nd	0.1	nd	nd	nd	13.7	9.5	3.5	36.3	661.1	2014.7	144.3	242.3	381.7	39.8	49.9	7.4	19.1	14.0	0.043	0.076	-
**86**	13.2	50.0	1.6	0.6	1.6	nd	nd	nd	nd	nd	201.5	13.6	5.7	9.0	120.9	162.0	34.6	30.9	55.2	2873.5	562.2	124.8	445.3	233.3	0.127	0.198	-
**87**	16.1	20.8	14.3	2.6	37.2	nd	nd	nd	nd	nd	1447.0	20.1	12.7	5.4	44.6	4.0	2.5	5.1	4.6	109.0	14.5	19.0	5.1	6.6	0.325	0.387	-
**88**	28.3	137.9	49.4	12.3	159.7	0.3	0.2	nd	0.1	nd	127.7	125.6	34.6	154.0	29.7	47.5	6.1	14.9	68.9	3448.1	4412.0	2704.2	5002.3	7808.1	<0.001	<0.001	S-BW; S-G; S-PC
**89**	0.1	<0.1	0.1	nd	<0.1	nd	nd	nd	nd	nd	10.0	0.8	0.1	0.4	56.9	2.7	0.5	5.5	4.3	139.0	45.8	66.0	857.7	430.9	0.041	0.095	-
**90**	nd	0.4	0.4	nd	0.3	nd	nd	nd	nd	nd	nd	0.1	nd	0.1	0.3	1.7	1.1	6.7	3.4	45.8	149.4	29.6	165.0	29.2	0.004	0.017	S-BW; S-G; S-PC
**91**	21.5	22.1	5.5	5.9	0.4	nd	nd	nd	nd	nd	8.1	2.4	3.7	7.4	1529.7	936.9	334.6	397.2	74.7	31.4	31.5	13.0	21.7	4.4	0.009	0.021	PC-BW; PC-G; S-PC
**92**	12.8	70.9	17.5	45.5	29.2	0.2	0.3	0.2	0.2	0.3	10.2	7.0	8.6	72.7	699.1	2149.6	294.6	619.2	858.0	26.5	31.4	13.3	37.2	22.9	0.003	0.01	PC-BW; PC-G; S-PC
**93**	56.2	119.4	20.7	8.1	315.5	0.7	0.7	nd	0.6	0.1	189.6	101.7	12.0	184.3	93.5	77.1	9.8	17.7	159.0	5113.8	4837.0	530.3	1557.1	12851.9	0.017	0.046	S-BW; S-G; S-PC
**94**	13.0	38.9	1.9	8.9	18.7	nd	nd	nd	nd	nd	12.0	6.5	4.8	53.2	679.0	961.2	67.6	412.2	639.2	41.8	25.5	6.2	23.0	22.6	<0.001	0.002	PC-BW; PC-G; S-PC
**95**	864.7	1253.2	96.3	7.6	1935.5	2.2	4.5	nd	1.1	3.6	5766.1	200.9	52.4	209.9	535.4	37.3	11.1	8.9	62.6	10790.0	975.5	2583.0	287.6	472.0	0.384	0.434	-
**96**	76.6	41.4	3.4	8.5	1.0	nd	<0.1	nd	nd	nd	19.6	3.8	4.3	7.8	4116.9	1373.3	173.7	438.3	57.8	209.0	24.9	7.8	20.6	7.0	0.118	0.145	-
**97**	121.4	2.2	0.3	0.9	4.0	nd	nd	nd	nd	0.5	1569.6	10.4	14.4	2.1	169.8	1.5	1.9	8.2	7.4	1147.9	10.4	11.4	15.0	21.9	0.546	0.555	-
**98**	156.9	35.0	19.6	0.6	50.3	0.2	0.2	nd	nd	0.4	462.3	6.1	4.0	3.3	70.2	3.8	0.4	11.0	9.4	1881.6	14.2	42.9	29.1	17.9	0.536	0.551	-
**99**	5.5	7.6	3.6	16.4	19.0	<0.1	nd	<0.1	nd	nd	4.4	3.9	9.2	49.3	663.2	318.0	261.8	1088.7	582.6	24.5	16.8	15.0	51.3	26.5	<0.001	0.001	PC-BW; PC-G; S-PC
**100**	6.1	32.2	82.8	134.9	10.0	0.3	0.4	0.4	0.2	0.3	6.8	7.2	73.0	55.4	174.5	349.8	5086.2	7383.7	147.6	13.4	26.6	140.2	229.6	8.1	0.087	0.122	-
**101**	1.7	7.1	28.8	0.1	4.1	nd	nd	nd	nd	nd	2.3	2.1	0.4	15.7	297.2	281.6	1491.4	21.3	187.1	8.5	22.8	96.1	7.8	8.2	0.093	0.124	-
**102**	2.2	46.0	0.3	26.3	0.4	<0.1	<0.1	nd	nd	nd	7.8	5.3	14.1	9.6	188.5	1149.1	62.5	1016.1	41.7	11.2	33.8	6.2	65.4	3.5	0.042	0.076	-
**103**	3.7	11.7	10.6	24.0	105.0	0.4	0.3	0.2	0.1	0.1	6.1	6.5	14.1	314.9	320.7	408.7	509.3	1407.5	3597.6	17.6	23.7	40.2	76.7	185.2	0.047	0.08	-
**104**	106.5	815.9	19.1	14.5	1201.8	0.3	1.7	nd	nd	nd	178.9	215.9	11.3	118.2	51.8	82.1	3.6	12.4	90.6	379.8	1241.2	94.4	358.6	642.5	0.149	0.22	-

	Body Walls					Coelomic fluid					Gonads				Pyloric caeca					Stomach					*p-value*	*q-value*	Tukey's HSD
N	BW#1	BW#2	BW#3	BW#4	BW#5	CF1	CF2	CF3	CF4	CF5	G#1	G#2	G#4	G#5	PC#1	PC#2	PC#3	PC#4	PC#5	S#1	S#2	S#3	S#4	S#5			
**105**	0.1	18.9	21.9	0.2	3.1	nd	nd	nd	nd	nd	8.0	29.3	3.4	5.8	3.2	18.4	11.3	8.4	9.1	161.2	1549.9	1123.5	754.0	360.3	0.001	0.007	S-BW; S-G; S-PC
**106**	10.2	35.5	30.5	5.2	67.1	<0.1	nd	nd	nd	nd	21.6	32.7	3.4	27.8	39.7	32.5	5.2	29.6	91.9	451.0	602.9	422.0	476.9	877.1	<0.001	<0.001	S-BW; S-G; S-PC
**107**	102.9	218.5	133.4	1121.5	1463.7	1.2	1.1	0.8	0.5	0.9	112.2	41.1	630.0	4784.8	5888.6	7274.8	6844.6	60500.2	44724.5	397.6	331.4	548.0	3371.8	2210.5	0.03	0.058	-
**108**	27.1	34.5	8.2	25.7	60.1	<0.1	0.1	nd	nd	0.1	18.0	6.3	15.2	175.6	1423.8	1014.0	401.4	1457.1	1659.8	87.5	31.4	29.6	65.9	78.3	<0.001	<0.001	PC-BW; PC-G; S-PC
**109**	481.1	446.1	76.0	91.3	182.1	0.9	1.2	0.6	0.5	0.4	146.2	38.4	48.4	621.8	20976.4	11403.6	3733.6	4661.0	4457.7	1388.0	261.8	176.4	233.9	321.9	0.005	0.015	PC-BW; PC-G; S-PC
**110**	130.4	215.3	58.9	6.1	255.5	0.6	1.0	nd	nd	0.7	1187.9	58.3	23.5	56.9	97.5	12.9	5.4	6.4	36.1	2984.0	485.5	499.1	98.0	478.6	0.197	0.269	-
**111**	19.9	286.2	417.6	360.5	437.8	0.3	1.2	nd	nd	nd	185.2	117.2	187.4	124.1	70.7	57.5	90.7	115.9	222.5	2257.3	1539.2	8349.9	7599.6	2541.8	0.002	0.01	S-BW; S-G; S-PC
**112**	73.4	34.9	17.4	8.2	133.6	0.2	0.2	nd	0.1	0.1	36.9	8.5	2.2	17.4	13.4	8.3	0.3	3.2	12.5	1202.6	868.1	87.0	122.2	996.0	0.004	0.017	S-BW; S-G; S-PC
**113**	203.0	84.4	4.4	47.4	2.4	0.1	0.2	<0.1	0.1	<0.1	50.5	11.3	24.6	18.4	4690.4	2423.0	258.3	2600.9	55.3	596.9	103.6	15.0	141.9	12.3	0.019	0.039	PC-BW; PC-G; S-PC
**114**	12.0	139.5	0.1	0.3	2.4	<0.1	0.2	nd	nd	nd	21.8	47.9	1.5	11.2	648.1	3925.5	10.3	18.9	106.2	41.0	137.5	2.8	25.4	5.7	0.301	0.317	-
**115**	14.7	31.3	27.8	51.3	44.5	0.1	0.1	<0.1	nd	<0.1	8.7	5.3	28.7	149.8	760.9	952.5	1494.4	3499.7	1729.7	39.5	47.0	105.9	199.5	82.7	0.001	0.003	PC-BW; PC-G; S-PC
**116**	650.5	3504.6	330.6	27.3	3271.5	2.2	6.2	nd	<0.1	2.2	333.2	191.2	12.0	122.5	75.8	114.0	8.9	5.1	81.2	1263.2	2469.3	892.8	171.8	1021.3	0.078	0.15	-
**117**	30.2	23.6	14.3	34.4	2.6	0.1	<0.1	<0.1	<0.1	<0.1	10.3	3.1	17.4	20.5	1773.3	911.6	720.8	1433.1	135.7	95.5	60.6	246.3	132.8	9.6	0.001	0.003	PC-BW; PC-G; S-PC
**118**	333.6	828.3	583.9	136.1	2275.8	1.0	1.0	<0.1	1.3	0.8	3792.8	4267.7	626.2	2579.2	3005.3	1715.8	326.9	539.8	7021.7	14585.0	4969.3	10475.0	8972.7	15859.4	<0.001	0.002	S-BW; S-G; S-PC
**119**	6.4	13.6	1.8	6.0	41.1	nd	nd	nd	nd	nd	2.1	1.0	2.0	143.3	268.8	302.4	65.8	314.4	1770.9	7.4	6.9	1.7	2.8	16.8	0.087	0.122	-
**120**	74.9	189.9	16.5	46.7	3.6	0.1	0.3	<0.1	<0.1	nd	33.9	17.7	24.3	22.2	4220.5	6049.3	911.9	3073.3	96.7	216.5	229.0	53.7	102.9	9.2	0.006	0.018	PC-BW; PC-G; S-PC
**121**	219.0	6582.6	83.6	109.6	8712.1	<0.1	0.3	nd	nd	nd	80.8	63.3	6.8	54.0	50.4	65.0	3.0	8.1	101.3	328.5	519.4	27372.1	36028.8	253.5	0.144	0.22	-
**122**	579.9	1634.5	5336.8	1523.3	3693.0	8.7	7.7	4.1	10.1	4.3	8143.0	4067.8	10983.5	4771.8	1763.9	2631.2	3245.4	6577.6	5454.2	48739.7	53931.2	284567.3	376034.2	103719.3	0.007	0.026	S-BW; S-G; S-PC
**123**	489.0	5479.4	2757.6	408.1	5097.8	2.7	9.0	0.1	0.6	0.8	162.2	133.3	44.3	311.6	24.0	39.8	17.6	8.3	124.1	222.0	518.7	700.3	75.5	152.2	0.009	0.03	G-BW; PC-BW; S-BW
**124**	17.8	102.0	2.9	8.4	8.3	<0.1	0.1	nd	<0.1	<0.1	16.8	10.7	5.6	28.4	1157.8	3852.9	174.7	593.2	295.3	81.5	181.8	12.3	30.3	18.1	0.079	0.116	-
**125**	25.8	49.9	18.3	19.4	34.3	0.1	0.1	nd	nd	nd	8.7	5.0	9.1	98.5	1433.3	1496.1	1074.6	1177.6	1218.6	83.2	69.8	344.4	792.7	59.5	<0.001	<0.001	PC-BW; PC-G; S-PC
**126**	18.8	69.0	22.1	41.1	167.2	0.1	0.1	<0.1	<0.1	<0.1	10.6	7.1	17.8	532.5	1000.0	2356.5	1179.8	2705.8	5356.8	72.1	105.4	26.3	33.6	288.0	0.001	0.006	PC-BW; PC-G; S-PC
**127**	128.4	86.6	5.8	26.0	3.3	0.1	0.2	<0.1	<0.1	nd	36.3	8.9	14.7	19.6	7760.8	3503.5	335.2	1578.7	123.4	312.8	147.6	14.7	25.7	13.4	0.057	0.094	-
**128**	433.1	4191.7	31.8	42.2	9053.9	3.4	13.4	nd	<0.1	0.3	2979.7	365.3	43.6	522.4	346.2	73.1	10.1	33.4	199.7	1310.9	437.1	69.0	94.8	301.4	0.256	0.329	-
**129**	21.3	40.2	36.4	8.1	80.9	0.5	0.2	<0.1	0.1	0.2	122.1	39.0	36.2	75.0	36.6	22.1	7.5	23.5	38.8	2727.1	1288.5	1743.8	3810.5	3817.9	<0.001	<0.001	S-BW; S-G; S-PC
**130**	429.2	1304.5	30.0	3.0	1508.2	0.2	0.1	nd	nd	<0.1	305.9	25.5	2.7	19.1	57.3	14.0	1.5	11.3	16.3	1420.5	310.8	90.7	37.6	111.7	0.189	0.264	-
**131**	42.9	80.0	88.5	235.4	93.5	0.1	0.1	0.1	<0.1	nd	27.2	21.6	101.6	346.9	1827.5	2467.6	3213.3	12148.3	2229.6	174.2	159.2	177.9	260.0	199.8	0.022	0.045	PC-BW; PC-G; S-PC
**132**	252.6	222.8	36.9	0.7	746.6	<0.1	0.1	nd	nd	nd	21675.3	489.9	174.0	189.7	1280.2	50.7	36.6	21.7	90.4	1944.6	146.3	428.8	95.9	186.7	0.323	0.387	-
**133**	19.7	26.7	10.4	48.4	0.3	<0.1	<0.1	<0.1	<0.1	nd	4.7	2.1	4.4	5.5	1320.0	1066.3	194.7	489.3	30.2	35.6	63.5	47.2	160.3	3.3	0.012	0.028	PC-BW; PC-G; S-PC
**134**	8.2	137.3	441.3	684.8	18.9	1.0	1.2	1.2	0.6	0.8	46.8	42.3	417.4	254.9	425.1	4246.4	17724.9	32643.9	475.9	43.4	400.0	1118.1	1348.5	37.6	0.082	0.118	-
**135**	547.4	416.2	433.3	115.2	674.2	4.6	4.2	1.8	5.7	3.6	5379.4	1840.9	1271.8	881.5	601.5	236.0	78.5	190.3	260.4	66353.2	37735.9	53881.5	102158.9	62721.9	<0.001	<0.001	S-BW; S-G; S-PC
**136**	148.1	203.5	141.3	351.8	529.3	1.1	1.0	0.8	0.6	0.6	90.5	34.0	230.0	1842.6	9716.0	8446.9	6865.8	20963.6	17103.3	359.1	584.5	564.4	1614.3	804.8	<0.001	<0.001	PC-BW; PC-G; S-PC
**137**	106.7	1254.5	158.8	8.9	1870.8	1.2	3.9	nd	nd	0.2	148.4	27.6	5.3	76.4	9.8	9.2	6.8	26.8	36.9	102.7	58.0	96.4	11.5	23.0	0.087	0.158	-
**138**	32.9	100.2	54.8	80.6	141.4	0.4	0.4	0.3	0.1	0.2	37.3	22.8	124.6	557.7	2017.6	4406.6	3134.4	4121.9	5374.4	103.0	187.1	34.4	55.6	231.4	<0.001	<0.001	PC-BW; PC-G; S-PC
**139**	1.1	0.8	16.6	44.5	8.7	0.2	0.2	0.1	0.1	0.1	0.9	0.6	1.5	7.3	123.5	60.0	1115.9	9287.4	356.8	33.0	9.6	373.1	661.3	49.6	0.316	0.33	-
**140**	364.5	4911.2	105.3	103.2	5453.0	1.6	5.2	0.2	0.5	0.8	996.0	1460.6	47.4	814.7	277.5	812.7	6.9	14.4	671.5	1988.3	8404.5	233.8	1640.3	5397.0	0.161	0.231	-

	Body Walls					Coelomic fluid					Gonads				Pyloric caeca					Stomach					*p-value*	*q-value*	Tukey's HSD
N	BW#1	BW#2	BW#3	BW#4	BW#5	CF1	CF2	CF3	CF4	CF5	G#1	G#2	G#4	G#5	PC#1	PC#2	PC#3	PC#4	PC#5	S#1	S#2	S#3	S#4	S#5			
**141**	20.0	26.5	10.4	47.2	3.5	0.1	<0.1	<0.1	<0.1	<0.1	6.9	2.2	19.4	14.3	129.9	89.8	570.2	1643.4	27.6	11.4	11.9	14.2	33.7	3.3	0.123	0.147	-
**142**	41.5	53.8	99.8	370.7	77.7	0.8	0.8	0.8	0.4	0.5	38.0	13.8	227.5	344.3	2423.3	1717.5	5516.0	25018.3	2415.6	271.7	110.4	298.2	499.6	168.8	0.105	0.135	-
**143**	355.4	672.4	10.9	8.7	3021.2	1.2	2.6	nd	nd	3.0	251.3	169.3	11.1	188.1	24.7	12.3	0.5	4.9	69.9	407.7	170.2	45.9	78.9	212.0	0.274	0.349	-
**144**	42.7	299.4	216.6	7.7	126.0	0.1	0.2	nd	<0.1	nd	30.3	35.6	3.1	8.8	2.5	14.7	6.8	1.2	11.4	36.2	356.2	2681.3	47.4	195.7	0.297	0.366	-
**145**	14.1	34.1	0.1	0.1	8.0	<0.1	<0.1	<0.1	<0.1	nd	17.5	8.3	0.9	22.7	790.5	1206.7	13.2	28.0	301.5	55.8	42.6	1.8	10.8	14.0	0.043	0.076	-
**146**	16.5	24.0	12.4	0.2	1.0	<0.1	0.1	<0.1	<0.1	<0.1	8.1	3.5	0.9	9.7	1124.5	996.5	666.4	23.0	112.3	29.0	68.3	23.0	7.0	6.2	0.007	0.019	PC-BW; PC-G; S-PC
**147**	5.2	55.3	38.8	24.9	1.2	<0.1	<0.1	nd	nd	nd	7.6	26.8	8.7	14.7	337.6	1820.1	1773.4	1273.3	62.9	17.4	80.8	123.8	72.4	5.7	0.003	0.01	PC-BW; PC-G; S-PC
**148**	23.3	14.5	10.7	22.6	3.6	<0.1	<0.1	<0.1	nd	nd	6.6	2.3	9.5	17.8	1321.4	515.3	602.9	1272.3	175.0	59.6	23.5	16.6	27.6	8.7	0.001	0.003	PC-BW; PC-G; S-PC
**149**	62.3	505.8	293.1	22.5	397.0	0.2	1.2	nd	<0.1	nd	175.1	17.9	7.5	20.8	9.8	4.5	1.4	0.1	11.5	126.6	47.8	242.3	16.2	17.8	0.034	0.082	-
**150**	540.3	1512.6	377.2	316.1	3502.2	4.4	5.0	1.2	4.9	4.8	2212.2	1126.0	122.8	934.6	194.2	398.7	11.2	25.9	489.8	25710.7	34145.3	3104.1	12623.3	66909.2	0.008	0.026	S-BW; S-G; S-PC
**151**	41.0	244.9	46.3	36.6	12.3	0.4	0.6	0.2	0.2	0.1	44.3	41.0	22.8	56.7	2543.9	8308.0	2693.6	2011.1	464.3	139.4	560.6	141.3	162.9	28.8	0.014	0.032	PC-BW; PC-G; S-PC
**152**	139.9	500.5	153.5	39.1	1166.6	1.0	0.5	0.1	1.0	0.1	2934.8	493.7	122.7	172.7	192.7	164.6	29.3	29.3	154.3	7144.0	5105.7	4667.3	9264.6	6723.2	<0.001	<0.001	S-BW; S-G; S-PC
**153**	165.1	93.4	57.8	92.8	3.6	0.4	0.4	0.2	0.2	0.1	56.6	14.3	50.4	40.5	10733.6	3850.3	3295.0	6150.0	120.7	614.8	308.4	422.2	344.8	23.4	0.005	0.015	PC-BW; PC-G; S-PC
**154**	58.7	153.6	176.8	325.3	526.7	0.9	0.8	0.7	0.4	0.5	52.6	34.5	183.9	1681.7	3398.5	5175.8	7696.1	19630.7	13505.1	233.6	287.7	591.9	1059.0	973.5	0.001	0.005	PC-BW; PC-G; S-PC
**155**	11.9	29.1	17.3	29.5	515.2	0.7	0.6	0.4	0.3	0.4	19.9	7.5	20.1	1413.2	675.5	1199.5	849.5	1737.0	15716.3	38.2	81.6	112.0	112.3	995.3	0.247	0.277	-
**156**	0.4	0.8	0.4	nd	4.5	nd	nd	nd	nd	nd	48.7	1.9	0.9	0.1	6.9	nd	nd	0.8	0.1	247.4	54.9	118.3	1236.8	245.8	0.079	0.15	-
**157**	3596.0	4270.7	247.7	112.9	10421.1	9.0	13.3	0.4	1.1	26.4	2984.1	471.9	121.6	489.3	214.3	86.0	22.1	29.8	200.5	5927.2	2898.0	3239.4	6855.4	4183.1	0.032	0.079	-
**158**	29.8	94.2	19.5	0.2	130.5	0.3	0.6	nd	nd	0.1	31.9	9.7	2.9	7.9	8.8	6.3	2.5	2.9	9.2	93.4	66.3	125.9	25.8	41.0	0.035	0.083	-
**159**	167.8	1285.7	1786.7	277.6	2053.0	<0.1	0.6	nd	nd	nd	511.7	368.1	63.7	278.2	65.5	55.8	32.0	17.1	163.0	507.3	415.7	493.3	152.9	254.2	0.015	0.046	G-BW; PC-BW; S-BW
**160**	18.0	190.2	955.1	54.5	87.6	0.6	0.7	0.1	0.4	<0.1	173.3	505.3	150.4	101.7	56.8	274.5	302.5	78.6	105.2	1753.7	8243.3	47948.8	10879.9	4370.2	0.085	0.157	-
**161**	0.6	9.5	231.5	70.7	18.3	0.9	0.6	0.3	0.3	0.2	78.0	20.1	1388.3	103.3	71.6	30.3	1200.9	1986.6	226.5	376.8	382.1	24757.8	4086.5	438.5	0.313	0.381	-
**162**	2.3	85.9	0.8	0.1	0.1	nd	0.1	nd	nd	nd	11.4	8.6	0.4	1.7	150.2	926.1	112.1	13.0	11.2	6.6	146.9	6.6	29.2	1.5	0.263	0.287	-
**163**	42.1	70.4	70.3	44.5	125.2	0.2	0.3	0.1	<0.1	0.1	25.5	15.2	29.5	352.8	2533.6	2803.6	3856.6	2849.6	2990.6	124.8	141.9	253.4	176.3	166.2	<0.001	<0.001	PC-BW; PC-G; S-PC
**164**	167.9	280.8	47.9	169.4	384.6	0.5	0.6	0.4	0.3	0.3	49.6	57.9	56.5	862.8	8078.0	7436.9	1775.2	8096.6	7645.3	705.7	592.2	211.9	605.0	711.0	<0.001	<0.001	PC-BW; PC-G; S-PC
**165**	164.8	238.3	34.7	183.8	28.8	0.4	0.6	0.1	0.2	0.1	68.9	45.7	96.3	135.0	8432.5	7242.8	1763.2	10733.0	732.7	625.5	267.9	119.1	502.8	63.6	0.002	0.009	PC-BW; PC-G; S-PC
**166**	889.8	704.5	13656.4	6416.2	1561.4	5.5	3.3	4.8	7.2	6.8	119.0	30.8	593.5	103.7	21.3	7.7	74.1	67.1	39.5	233.4	180.3	4437.4	217.6	167.9	0.103	0.173	-
**167**	267.2	42.8	0.8	11.7	3.8	0.2	0.2	<0.1	0.1	nd	81.6	14.4	6.8	11.8	13744.3	1401.4	100.4	432.7	84.4	744.3	55.6	8.8	34.2	19.8	0.338	0.349	-
**168**	77.5	430.1	2486.5	565.6	450.0	0.1	nd	nd	0.1	nd	151.0	48.0	51.9	51.3	18.2	17.5	13.3	9.8	20.9	147.9	300.2	1233.3	182.4	149.4	0.147	0.22	-
**169**	9.9	26.1	122.6	505.7	1067.1	1.7	1.5	1.1	0.7	1.0	51.9	19.1	327.9	3377.1	846.6	1159.7	6230.3	30397.1	25925.6	61.9	91.7	539.6	1864.8	1938.1	0.047	0.08	-
**170**	7.9	26.0	55.5	5.1	11.6	nd	<0.1	nd	nd	nd	37.8	31.3	11.9	6.1	17.0	14.9	16.7	5.7	5.5	591.4	401.2	2609.1	568.3	189.6	0.043	0.099	-
**171**	177.1	283.2	50.0	173.3	398.1	1.0	1.2	0.7	0.6	0.7	78.2	70.4	147.9	1383.3	3902.7	3400.2	7949.7	11623.8	10862.9	279.4	190.6	549.5	714.0	657.8	<0.001	0.001	PC-BW; PC-G; S-PC
**172**	15.0	7.1	11.9	25.1	31.6	<0.1	<0.1	<0.1	<0.1	<0.1	8.4	5.2	19.7	119.8	1300.1	297.7	723.3	1797.0	1141.8	325.6	32.6	68.7	111.8	79.5	<0.001	0.001	PC-BW; PC-G; S-PC
**173**	2135.4	1439.0	32.8	31.3	5636.7	3.7	4.8	nd	<0.1	10.6	8447.0	369.6	81.4	327.1	576.8	51.1	6.9	9.3	127.1	1971.4	406.7	126.3	128.1	477.5	0.439	0.477	-
**174**	0.6	5.3	0.7	0.6	1.2	0.6	nd	nd	0.2	nd	14.1	7.0	5.6	27.7	86.6	50.9	41.6	195.2	84.8	909.9	710.8	110.2	1712.5	651.0	0.002	0.009	S-BW; S-G; S-PC
**175**	48.4	111.6	204.6	79.5	265.5	0.7	0.5	nd	0.4	<0.1	613.3	257.2	242.7	306.7	277.7	141.4	79.0	239.8	387.8	4657.0	2556.3	8932.2	12245.8	9725.7	<0.001	0.001	S-BW; S-G; S-PC
**176**	43.1	66.9	2283.0	503.0	133.0	1.0	0.6	0.1	0.4	<0.1	162.3	11.4	78.3	21.3	16.7	1.3	17.0	12.3	7.5	398.8	82.3	4341.6	218.6	178.5	0.424	0.465	-

	Body Walls					Coelomic fluid					Gonads				Pyloric caeca					Stomach					*p-value*	*q-value*	Tukey's HSD
N	BW#1	BW#2	BW#3	BW#4	BW#5	CF1	CF2	CF3	CF4	CF5	G#1	G#2	G#4	G#5	PC#1	PC#2	PC#3	PC#4	PC#5	S#1	S#2	S#3	S#4	S#5			
**177**	130.1	968.7	278.5	19.4	1419.9	0.3	1.9	nd	nd	nd	1522.4	112.7	24.8	91.2	64.2	23.3	4.1	3.2	54.0	470.9	147.0	176.7	29.5	45.3	0.29	0.361	-
**178**	1.8	0.7	0.5	10.5	10.2	1.1	0.2	0.1	0.4	0.1	28.6	8.4	9.5	54.8	197.5	106.8	94.9	505.1	193.7	1474.2	900.9	162.4	1728.2	852.3	0.001	0.004	S-BW; S-G; S-PC
**179**	176.4	517.7	39.1	116.6	532.1	1.6	2.1	1.0	0.7	0.9	129.8	70.0	99.4	1845.8	10130.1	14976.0	2479.3	7496.3	19375.3	639.9	806.0	219.7	619.2	1123.6	<0.001	0.002	PC-BW; PC-G; S-PC
**180**	24.5	18.6	66.0	29.8	116.3	nd	nd	nd	0.1	nd	2316.3	209.7	140.8	235.4	169.5	94.4	22.6	94.1	94.6	2643.2	2111.4	4624.6	11194.8	3774.3	0.004	0.017	S-BW; S-G; S-PC
**181**	11.4	99.4	108.1	40.8	7.8	0.2	0.3	0.2	<0.1	<0.1	17.8	17.5	25.2	27.0	761.0	3737.7	5788.2	2598.7	133.8	37.6	179.3	280.1	148.0	9.2	0.009	0.021	PC-BW; PC-G; S-PC
**182**	2497.1	4167.9	983.3	627.0	8925.0	10.2	12.7	2.1	7.5	14.0	4003.8	1902.6	165.7	1779.7	714.0	826.4	43.4	83.4	1405.5	48534.8	47305.1	1990.1	9162.0	75914.8	0.008	0.026	S-BW; S-G; S-PC
**183**	47.6	60.5	4.2	12.5	0.1	<0.1	0.1	nd	<0.1	nd	16.9	10.0	7.6	7.1	2334.3	1980.8	231.1	834.3	27.1	146.8	106.2	17.8	61.7	5.0	0.017	0.036	PC-BW; PC-G; S-PC
**184**	3.1	22.7	115.5	56.2	35.5	0.4	0.5	0.4	0.2	0.2	6.1	8.1	35.8	107.8	96.5	349.9	5133.3	3205.6	1340.6	10.4	50.5	526.7	205.6	86.4	0.033	0.063	-
**185**	4.9	3.6	37.5	20.2	204.6	0.1	<0.1	<0.1	nd	<0.1	10.3	5.6	15.2	692.9	404.8	300.9	2095.2	1379.7	8216.7	49.2	36.8	161.3	94.9	368.0	0.114	0.145	-
**186**	4.4	7.2	14.1	0.8	12.3	7.1	8.5	7.6	6.0	7.7	2342.1	80.1	60.7	50.4	110.1	25.1	22.8	47.1	44.7	2829.6	974.5	1702.8	9603.1	3777.1	0.016	0.046	S-BW; S-G; S-PC
**187**	46.9	9.9	22.2	16.6	29.2	0.1	<0.1	nd	<0.1	nd	7138.4	62.3	69.5	43.2	360.9	27.9	6.8	17.4	18.1	1998.2	120.8	798.9	1544.7	185.8	0.353	0.412	-
**188**	2.8	46.6	24.6	17.9	369.6	1.1	1.1	0.8	0.4	0.7	20.3	18.2	24.3	1212.6	146.5	1811.8	823.7	845.5	13910.6	11.2	166.5	141.0	121.4	811.8	0.268	0.289	-
**189**	31.4	57.0	11.9	12.2	9.3	0.1	0.2	nd	nd	nd	19.3	9.7	9.0	32.8	2229.9	2326.5	659.8	834.3	385.9	100.4	106.9	37.8	64.7	19.3	0.002	0.006	PC-BW; PC-G; S-PC
**190**	819.0	1272.3	694.4	231.8	763.6	2.5	3.4	nd	0.9	2.0	16433.3	253.1	226.9	96.1	166.1	25.1	4.8	6.6	30.2	8230.7	921.5	428.3	222.5	922.7	0.454	0.489	-
**191**	0.5	83.0	3.3	0.4	62.1	<0.1	0.5	nd	nd	nd	46.5	16.5	<0.1	7.1	0.1	2.5	nd	0.1	4.8	33.7	57.6	92.9	32.8	17.0	0.101	0.172	-
**192**	26.1	18.1	620.7	198.9	44.7	0.3	0.1	nd	nd	nd	8.2	0.3	18.2	3.5	0.1	nd	5.2	6.6	0.9	9.7	6.0	118.8	7.6	6.1	0.166	0.235	-
**193**	89.8	48.0	477.0	183.9	89.2	0.7	0.1	nd	nd	0.1	31.6	1.0	18.4	5.9	2.8	nd	1.6	1.9	0.8	35.3	15.3	321.8	10.5	5.6	0.11	0.177	-
**194**	65.1	558.5	473.1	26.5	351.7	0.9	1.7	nd	nd	nd	8.3	10.0	2.5	16.6	0.1	3.0	2.7	6.6	10.9	15.1	21.2	98.4	4.9	12.0	0.006	0.024	G-BW; PC-BW; S-BW
**195**	653.1	1543.8	4173.5	1751.9	1557.2	2.6	2.7	0.5	1.5	2.1	458.7	110.4	230.8	112.4	22.2	9.8	8.8	17.0	14.9	421.6	407.3	2181.8	368.9	352.3	0.009	0.029	G-BW; PC-BW
**196**	19.8	32.8	230.7	530.9	863.3	1.6	1.0	0.9	0.5	0.7	45.3	23.3	298.1	2944.5	1540.8	1460.9	11699.2	33181.6	25318.7	119.1	135.0	1016.8	1905.3	2044.4	0.022	0.045	PC-BW; PC-G; S-PC
**197**	59.1	15.2	2.3	1.6	4.8	0.1	<0.1	nd	<0.1	nd	15.7	3.1	0.5	13.0	3978.5	620.8	52.6	35.1	113.8	228.2	23.6	4.3	7.9	18.2	0.289	0.308	-
**198**	157.3	122.3	5574.0	3952.7	551.7	0.2	nd	nd	nd	nd	255.1	56.0	443.0	91.1	43.3	9.7	173.6	268.5	58.9	190.5	59.0	744.3	100.0	69.6	0.097	0.171	-
**199**	16.1	3.5	2.2	1.1	1.1	4.4	4.2	4.1	3.1	4.1	175.3	22.3	18.9	30.0	226.6	25.1	14.4	38.7	38.1	4126.3	1289.7	1714.1	17776.5	11203.0	0.017	0.046	S-BW; S-G; S-PC
**200**	93.8	249.6	2325.5	2064.5	430.0	0.9	nd	<0.1	1.2	nd	483.4	70.5	614.2	116.5	54.2	19.1	164.5	319.7	51.5	409.4	722.2	8208.9	1435.2	613.8	0.281	0.354	-
**201**	7.2	6.5	1075.1	527.6	26.7	0.1	nd	0.1	0.1	<0.1	5.0	0.9	56.7	10.2	0.3	0.1	3.2	7.5	2.0	55.7	19.7	351.9	30.3	46.5	0.212	0.283	-
**202**	14.5	12.8	1.2	0.1	47.5	<0.1	nd	nd	nd	nd	7.3	2.7	2.5	175.2	1195.2	633.9	48.2	211.7	2531.8	66.8	40.5	9.2	22.4	97.6	0.039	0.072	-
**203**	368.5	263.8	5114.3	1743.7	277.9	1.6	0.1	1.3	1.9	1.5	403.2	86.5	621.5	78.3	22.6	5.1	24.3	34.3	10.7	1068.4	783.4	11283.8	958.3	550.5	0.335	0.395	-
**204**	0.6	1.8	9.4	78.1	5.6	<0.1	<0.1	nd	nd	nd	3.6	1.1	44.6	45.1	32.9	56.6	448.3	5233.9	222.9	3.7	5.5	44.9	324.4	13.1	0.342	0.35	-
**205**	1597.0	1008.3	795.8	359.8	1721.4	22.3	20.4	17.4	24.6	19.7	30876.2	3304.3	2029.5	4245.0	13186.6	4638.6	2979.9	9927.2	7953.1	42662.0	23410.4	52198.3	77821.2	30560.5	<0.001	0.002	S-BW; S-G; S-PC
**206**	16.4	75.5	547.4	799.2	73.4	nd	nd	nd	<0.1	nd	42.3	17.7	189.5	37.8	10.4	3.7	32.4	63.2	11.2	77.2	75.7	544.1	94.7	94.3	0.223	0.294	-
**207**	675.3	471.0	774.9	149.1	319.1	12.3	14.0	13.3	6.8	8.2	385.9	62.9	61.6	107.3	196.6	300.5	457.0	274.4	372.8	446.3	322.6	775.6	1042.3	735.4	0.017	0.046	S-G; S-PC

BW: body wall, G: gonads, PC: pyloric caeca, S: stomach; nd: not detected
